# Explosive dissolution and trapping of block copolymer seed crystallites

**DOI:** 10.1038/s41467-018-03528-x

**Published:** 2018-03-20

**Authors:** Gerald Guerin, Paul A. Rupar, Ian Manners, Mitchell A. Winnik

**Affiliations:** 1 Department of Chemistry, University of Toronto, 80 St. George Street, Toronto, ON M5S 3H6 Canada; 2 School of Chemistry, University of Bristol, Bristol, BS8 1TS UK; 3 Present Address: Department of Chemistry, University of Alabama, Tuscaloosa, AL 35487 USA

## Abstract

Enhanced control over crystallization-driven self-assembly (CDSA) of coil-crystalline block copolymers has led to the formation of intricate structures with well-defined morphology and dimensions. While approaches to build those sophisticated structures may strongly differ from each other, they all share a key cornerstone: a polymer crystallite. Here we report a trapping technique that enables tracking of the change in length of one-dimensional (1D) polymer crystallites as they are annealed in solution at different temperatures. Using the similarities between 1D polymeric micelles and bottle-brush polymers, we developed a model explaining how the dissolving crystallites reach a critical size independent of the annealing temperature, and then explode in a cooperative process involving the remaining polymer chains of the crystallites. This model also allows us to demonstrate the role of the distribution in seed core crystallinity on the dissolution of the crystallites.

## Abstract (web summary)

The study of the dissolution of polymer crystals is a challenging task. Here the authors use crystallization-driven self-assembly of coil-crystalline block copolymers as a trapping technique to track the change in length of 1D seed crystallites during annealing.

## Introduction

Crystallization of coil-crystalline block copolymers (BCPs) has proven to be an extremely versatile approach to prepare polymer crystals with controlled morphologies^1–11^, and has generated a broad and growing interest in the polymer community^12,13^. The ability of these BCPs to form micelles with an organic or organometallic core opens the possibility of a broad range of applications such as, nanomotors^14,15^, model systems for biological structures^16^, nanocarriers for gene delivery^17,18^ or semi-conducting nanowires^19^. Among these BCPs, those with a polyferrocenyldimethylsilane (PFS) core-forming block are the most intensely studied, because they can be used to prepare highly sophisticated structures, such as biomorphic supermicelles and 2D crystals, via stepwise hierarchical assembly^20–24^.

Fundamental studies of these core-crystalline micelles can deepen our general understanding of polymer crystallization and dissolution. For this purpose, we designed seed trapping experiments in solution that gave us information about the dissolution of polymer crystals as function of temperature. Here, seed trapping refers to the act of capturing a BCP crystal in a transient state with a large excess of free chains (unimer) of a second BCP with the same crystalline block but a different corona-forming block. The key features of our experiments reside (i) in the nature of the polymers studied, since PFS BCPs enable a high level of control over the dimensions of core-crystalline micelles formed in solution, and (ii) in the size of the seed crystallites we examine. In contrast with most polymer crystals studied so far, the crystallites we studied were one-dimensional, less than 50 nm long, and contained a very small number of BCP molecules (ca. 100). In this way, one could capture crucial events that occur during the early stages of the dissolution of polymer crystals. To follow the dissolution process, we heat solutions of short PFS_53_-*b*-PI_637_ (PI = polyisoprene; the subscripts refer to the mean degrees of polymerization) crystallites (seeds) in decane at different temperatures. We then trap the surviving crystallites by adding a large excess of PFS_60_-*b*-PDMS_660_ (PDMS = polydimethylsiloxane) unimer dissolved in hot decane. Upon cooling, the two different PFS BCPs compete for seeded growth on the surviving crystallites to form M_(PFS-PI)_-*r*-M_(PFS-PDMS)_-*b*-M_(PFS-PI)_-*b*-M_(PFS-PDMS)_-*r*-M_(PFS-PI)_ triblock co-micelles. By selectively staining the PI block^25,26^, we can measure the length of the surviving crystallites by TEM. We find that BCP dissolution occurs until the surviving crystallites reach a critical length, and then they dissolve in a process involving a cooperative and concerted motion of all the remaining chains. More important, we observe that the critical length does not depend on the annealing temperature.

## Results

### Seed dissolution experiments

Little is known about the dissolution of polymeric crystals. Mallapragada and Peppas examined the dissolution of thin semi-crystalline films of poly(vinyl alcohol) in water^27^. They observed film swelling accompanied by a decrease in crystallinity followed by dissolution of the amorphous domains. Barham and Sadler studied the melting of polyethylene crystals by small-angle neutron scattering^28^. Their results pointed toward an extremely rapid change of the polymer chain conformation that could only be explained by the cooperative motions of the folded chains, a process that de Gennes referred to as a “conformational explosion” upon melting^29^.

**Fig. 1:**
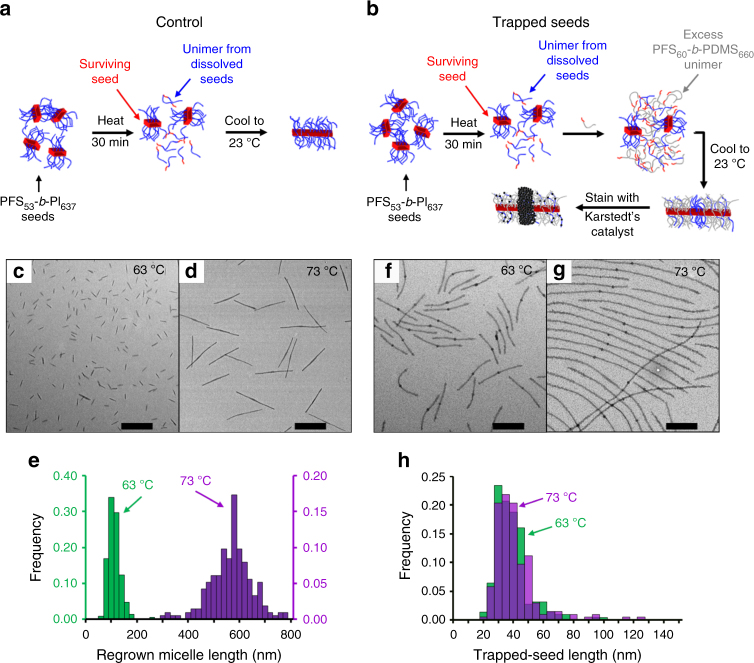
Investigation of crystallite dissolution by seed trapping. Schematic diagram describing **a** the control and **b** the seed trapping experiments performed to study the dissolution of PFS_53_-*b*-PI_637_ crystallites heated for 30 min in decane at different temperatures. In this scheme we use a color code to represent different chemical species: red represents polyferrocenyldimethylsilane (PFS) (either as the PFS block of a unimer or the crystalline core of a micelle), blue represents polyisoprene (PI), gray represents polydimethylsiloxane (PDMS), while the black spheres represent the platinum nanoparticles from Karstedt’s catalyst used to stain PI. **c** and **d** TEM micrographs of PFS_53_-*b*-PI_637_ micelles obtained by heating PFS_53_-*b*-PI_637_ seeds in decane for 30 minutes at **c** 63 °C, and **d** 73 °C, and cooling them to room temperature. **e** Length distribution histograms of the micelles shown in (**c**) (63 °C, green columns), and (**d**) (73 °C, purple columns). TEM micrographs of triblock comicelles obtained by heating PFS_53_-*b*-PI_637_ seeds 25 minutes at **f** 63 °C and **g** 73 °C then adding PFS_60_-*b*-PDMS_660_ unimer, and after five more minutes, letting the solution cool to room temperature. **h** Length distribution histograms of the surviving PFS_53_-*b*-PI_637_ seeds after the sample was annealed at 63 °C (green columns) and 73 °C (purple columns). Samples **f** and **g** were trapped with excess of PFS_60_-PDMS_660_ unimer, and stained with Karstedt’s catalyst to highlight the PI rich regions. Scale bars, 500 nm

To gain more information, we investigated the dissolution of short PFS_53_-*b*-PI_637_ crystallites in decane at different temperatures. These crystallites were formed by sonicating a solution of long PFS_53_-*b*-PI_637_ micelles suspended in decane. We explored 12 annealing temperatures ranging from 30 to 77 °C. For each annealing temperature, *T*_a_, we prepared two identical solutions of PFS_53_-*b*-PI_637_ seed crystallites in decane. One served as a control. It was removed from the oil bath after 30 min (Fig. 1a). For the second solution, the seeds were trapped after being heated for 25 min by adding a 5-fold excess of PFS_60_-*b*-PDMS_660_ unimer to the seed solution, which was then removed from the oil bath to cool to 23 °C (Fig. 1b). We monitored the number average length of the trapped seeds annealed at *T*_a_ for 30 min_,_ and the number average length of the micelles obtained from control experiments annealed in the same way and then cooled to 23 °C, *L*_mic_(*T*_a_). For each annealing temperature, we traced more than 200 micelles or stained trapped seeds to evaluate their number average length and standard error of the mean, s.e.m., obtained with a 99% confidence interval (Supplementary Methods).

We first ascertained that our trapping procedure did not affect the length distribution of the seeds (see Methods section) by adding an excess of PFS_60_-*b*-PDMS_660_ unimer to a solution of PFS_53_-*b*-PI_637_ crystallites at 23 °C (see Supplementary Fig. 1). The mean length and length distribution of the PFS_53_-*b*-PI_637_ seeds themselves (number average length, *L*_n_ = 43.5 ± 2.3 nm, Supplementary Fig. 1a) was slightly longer (ca. 3 nm) than that of the PFS_53_-*b*-PI_637_ crystallites trapped by PFS-*b*-PDMS (*L*_n_ = 40.9 ± 2.7 nm, Supplementary Fig. 1b). This small difference suggests that the staining procedure induced a contraction of the PI corona, leading to an underestimation of the length of the stained seeds. To verify this assumption, we compared the number average length of the stained seeds, as measured by TEM, to that of the untrapped seeds for annealing temperatures lower than 50 °C, where seed dissolution remains negligible^30,31^ (Supplementary Fig. 2). We found that at these annealing temperatures, the number average length of the stained seeds was systematically shorter than that of the untrapped seeds (control experiments) by ca. 3.5 nm. We thus define the length of the trapped seeds as the length of the stained seeds measured by TEM plus 3.5 nm. Supplementary Figure 3a shows the evolution of the average length of the trapped seeds, *L*_ts_(*T*_a_), as well as *L*_mic_(*T*_a_) as a function of the annealing temperature, for *T*_a_ ≤ 55 °C. The histograms of the lengths of the untrapped seeds, as well as those of the corrected lengths of the trapped seeds at *T*_a_ = 23, 30, 40, and 50 °C are shown in Supplementary Fig. 3b–e.

From knowledge of *L*_ts_(*T*_a_) and *L*_mic_(*T*_a_), we evaluated the extent of seed dissolution by calculating the apparent mass percentage of seeds (*%m*_ts_*(T*_*a*_)) that survived the annealing procedure (for the development of equation 1, see “Calculation of the mass percentage of seeds that survived the annealing procedure, %*m*_ts_(*T*_a_)” in the Supplementary Discussion):

1 $$\% m_{{\mathrm{ts}}}(T_{\mathrm{a}}) = \frac{{L_{\rm ts}(T_{\mathrm{a}})}}{{L_{\mathrm{mic}}(T_{\mathrm{a}})}} \times 100$$

Figure 1c, d presents TEM images obtained from the control experiments carried out at 63 °C (Fig. 1c) and 73 °C (Fig. 1d). These experiments show a typical self-seeding behavior^32–35^: the micelles are uniform in length, and their length increased with annealing temperature (*L*_mic_(63°) = 102 nm, and *L*_mic_(73°) = 558 nm) (Fig. 1e). This result indicates that fewer PFS_53_-*b*-PI_637_ seeds survived when the annealing temperature was increased.

The results obtained from the corresponding trapped seed solutions are presented in Figure 1f (63 °C) and 1g (73 °C). The addition of PFS_60_-*b*-PDMS_660_ unimer in excess led to micelles that were substantially longer than those seen in Fig. 1c, d. The staining protocol was very effective at delineating the surviving seed portion of the triblock comicelles. The end blocks of these M_(PFS-PI)_-*r*-M_(PFS-PDMS)_-*b*-M_(PFS-PI)_-*b*-M_(PFS-PDMS)_-*r*-M_(PFS-PI)_ co-micelles did not show any signs of PI/PDMS corona chain segregation, consistent with a previous study that examined the crystallization-driven co-assembly and seeded growth of mixtures of PFS-*b*-PI and PFS-*b*-PDMS^36^.

In contrast to the evolution of the length of the micelles regrown in the control experiments (Fig. 1c–e), the lengths of the Pt-stained trapped seeds (Fig. 1f, g) were similar at both annealing temperatures (*L*_ts_(63°) = 36.6 ± 2.0 nm and *L*_ts_ (73°) = 38.5 ± 2.6 nm), and were characterized by similar length distributions (Fig. 1h).

**Fig. 2:**
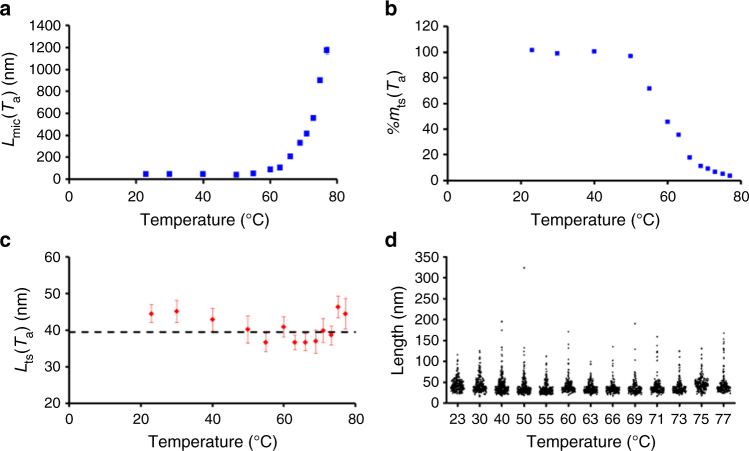
Effect of the dissolution temperature on the length and number of surviving seeds. **a** Evolution of the number average length of the PFS_53_-*b*-PI_637_ seed crystallites regrown at 23 °C as a function of annealing temperature (control experiment). **b** Evolution of the apparent mass percentage of surviving seeds as a function of annealing temperature. **c** Evolution of the length of the surviving seeds as a function of annealing temperature. The horizontal dashed line corresponds to the average length of the trapped seeds (averaged over the whole annealing temperature range). Error bars correspond to the s.e.m. of the length distributions determined by tracing more than 200 stained trapped seeds for each annealing temperature (see Supplementary Methods). **d** Length distributions of the surviving seeds at each annealing temperature. The surviving seeds were trapped by the addition of PFS_60_-*b*-PDMS_660_ prior to cooling, and stained with Karstedt’s catalyst

For all annealing temperatures, we determined *L*_mic_(*T*_a_), *%m*_ts_(*T*_a_), and *L*_ts_(*T*_a_) (Fig. 2a–c). Below 50 °C, *%m*_ts_(*T*_a_) remained constant (Fig. 2b, and Supplementary Table 1), while *L*_mic_(*T*_a_) decreased slightly from *L*_mic_(23°) = 43.5 ± 2.2 nm to *L*_mic_(50°) = 41.2 ± 3.0 nm (Fig. 2a, and Supplementary Table 1), and *L*_ts_(*T*_a_) decreased from *L*_ts_(23°) = 44.4 ± 2.7 nm to *L*_ts_(40°) = 40.1 ± 3.8 nm (Fig. 2c, and Supplementary Table 2). This result suggests that the seeds did not dissolve at these mild temperatures, but underwent some fragmentation^30,31^, as confirmed by the similarities between the length distributions of the trapped seeds and that of the seeds in the control experiments at these temperatures (Supplementary Fig. 3).

For *T*_a_ > 50 °C, *L*_mic_(*T*_a_) increased monotonically with increasing temperature from *L*_mic_(50°) = 41.2 ± 3.0 nm to *L*_mic_(77°) = 1173 ± 34 nm (Fig. 2a, and Supplementary Table 1), while *%m*_ts_(*T*_a_) decreased monotonically to reach a value of *%m*_ts_(77°) = 4%. TEM images of the micelles obtained in the seed trapping experiments are presented in Supplementary Figs. 4–6. Although the micelles grew longer as the annealing temperature increased, the dimensions of the Pt-stained seed portion remained unchanged. The most extreme example is shown in Supplementary Fig. 7 for *T*_a_ = 77 °C. While low-magnification images are necessary to observe the overall micelles (~5 μm long), their trapped-seed portion can only be resolved at higher magnification. By examining multiple images, we obtained the length distribution of the trapped seeds (Fig. 2d) and constructed corresponding histograms (see Supplementary Figs 8–9).

The evolution of *L*_ts_(*T*_a_) as a function of *T*_a_ (Fig. 2c, d) is remarkable. *L*_ts_(*T*_a_) is independent of both *T*_a_ and *%m*_ts_(*T*_a_), and the average length of the trapped seeds (averaged over the whole annealing temperature range), *L*_ts,av_ ≈ 39.5 nm, is only slightly shorter than the trapped starting seeds at room temperature, *L*_ts_(23°) = 44.4 ± 2.7 nm. In addition, over the range of annealing temperatures investigated, including at 77 °C where most of the seeds have dissolved (*%m*_ts_(77°) = 4%), there was only a negligible number of trapped seeds shorter than 20 nm.

One would anticipate that the seeds would dissolve from their ends, gradually shifting the trapped seed distributions to lower values as the annealing temperature increased. The absence of trapped seeds shorter than 20 nm, and the similarity between the trapped seeds histograms obtained at different annealing temperatures (Fig. 1h and Supplementary Figs 8–9) were thus highly surprising. Since every micelle has a Pt-stained seed and none with *L*_ts,av_ ≤ 20 nm, we conclude that these short seed dissolve by a cooperative process, involving a conformational explosion of PFS polymer molecules in the semicrystalline micelle core.

If the seeds dissolve from their ends, one would also expect to see a decrease of their length as a function of annealing time. To test this idea, we performed an isothermal kinetic study of micelle dissolution for longer annealing times, *t*_a_ = 100, 420, 1200, and 2640 min at an annealing temperature of *T*_a_ = 75 °C. These experiments were carried out one year after the dissolution experiments described above, using the same mother solution of PFS_53_-*b*-PI_637_ seeds in decane that was carefully stored in a sealed container at 23 °C. Over this storage time, the size and concentration of seed crystallites remained constant (Supplementary Fig. 10). Micelles regrown from the aged seeds after annealing at 75 °C, however, were much shorter than those obtained from the freshly prepared solution of PFS_53_-*b*-PI_637_ seeds in decane (Supplementary Fig. 11). They gave *L*_mic_(*T*_a_) values ranging from 55 to 65 nm, close to that obtained from a freshly prepared seed sample annealed at 55 °C (Supplementary Fig. 12). This difference in behavior indicates that the seeds aged at 23 °C for one year were much more stable toward dissolution than the freshly prepared solution. We attribute the enhanced robustness of the year-old seeds to an increase in the crystallinity of the 1D PFS core. While the fraction of surviving seeds for the year-old sample was higher than that of the fresh sample, the number average lengths and length distributions of the trapped seeds in both experiments were similar (Supplementary Figs 13 and 14). This result provides a clear indication that there is no systematic decrease in the length of the trapped seeds with prolonged annealing^34^, as first observed by Blundell and Keller for self-seeded polyethylene single crystals in xylene^37^.

These experiments provide important insights into the fragmentation and dissolution of the micelle crystallite seeds. They show, for example, that the extra aging time did not affect the mechanism of dissolution. They also show that seed dissolution at each temperature must be a relatively rapid process, since when the seeds survive, they do not dissolve further^37^.

To explain this phenomenon, we turned to the numerous studies of the scission of bottlebrush polymers induced by side chain repulsion^38–42^. In this context, we considered the effect of corona chain repulsion on the stability of the seed crystallites in terms of their free energy. The free energy of micelles with a crystalline core and a solvent swollen corona formed under thermodynamic equilibrium is equal to the sum of the free energy of crystalline core, Δ*F*_core_, that of the interface between the core and the solvent (the corona being ignored), Δ*F*_s_^43^, and that of the corona chain, Δ*F*_corona_.

### Free energy of the core

We have shown in a previous publication^44^ that the PFS core of micelles prepared by crystallization-driven self-assembly (CDSA) can be pictured as a crystalline layer sandwiched between two layers of PFS in the amorphous state, in which each amorphous layer contains *α* repeat units per fold. We can thus modify the equation of the free energy of a crystalline block^45^ to account for these amorphous layers:

2 $${\mathrm{\Delta }}F_{{\mathrm{core}}}\, \approx \,\varepsilon _1N_{{\mathrm{core}}} + \alpha \,\delta \varepsilon \;n_{{\mathrm{core}}}$$

where *ε*_1_ is the energy of a unit in the crystalline state, *n*_core_, the number of folds per block, *N*_core_, the number of units of the core forming block, and δ*ε*, the excess energy between an amorphous and a crystalline unit. The value *α* = 1 leads to the equation originally developed by Birshtein and Zhulina^45^, where a kink in fold formation contains only one amorphous unit. α should be smaller than *N*_core_/(*n*_core_ + 1), since the core must contain more than one crystalline unit per fold.

### Interface free energy

We assume that only one unit per fold is located at the surface of the layer (the other amorphous units being buried inside the amorphous layers), which leads to^45^:

3 $${\mathrm{\Delta }}F_{\mathrm{s}}\, = \,\frac{{a\,N_{{\mathrm{core}}}}}{{t_1}}\left( {{\mathrm{\varphi }} + \,\delta \varepsilon } \right)$$

with *t*_1,_ the half thickness of the crystalline core, *a*, the size of a monomer, and *φ*, the surface tension coefficient.

### Free energy of the corona

There is an important similarity between the corona chains of a 1D micelle, and the side chains of a bottle-brush polymer. Their dense packing induces strong mutual repulsions between neighboring chains, and forces the chains to stretch further away from their grafting points. As a consequence, the free energy of the corona chains, like those of the bottle-brush side chains, can be described as the sum of the free energy of stretching of the chains, Δ*F*_elast_, with that of the chains in semidilute solution, Δ*F*_conc_^46^.

4 $${\mathrm{\Delta }}F_{{\mathrm{corona}}} = {\mathrm{\Delta }}F_{{\mathrm{conc}}} + {\mathrm{\Delta }}F_{{\mathrm{elast}}}\, \approx \,N_{{\mathrm{corona}}}^{3/8}\,\tau ^{1/8}N_{{\mathrm{agg,L}}}^{5/8}$$

Here *N*_agg, L_ is the number of BCP molecules per unit length, *τ* represents the quality of the solvent as a function of the temperature of the solution, *T* (*τ* = (*T*-*θ*)/T), with *θ*, the theta temperature. Moreover, the thickness of the corona, D, is given by:

5 $$D\, \approx \,N_{{\mathrm{corona}}}^{3/4}\,\tau ^{1/4}N_{{\mathrm{agg,L}}}^{1/4}$$

When *T* increases, (i) the amorphous layers of the PFS core become thicker, decreasing the crystallinity of the seeds (eq. 2), and (ii) decane becomes an even better solvent for the PI corona chains, stretching the corona chains (eq. 5) and increasing their free energy (eq. 4). These concomitant phenomena affect both the core and the corona of the seeds, inducing their dissolution.

### Seed fragmentation and critical length

The concept of a superblob was developed by Birshtein and Zhulina to evaluate the local rigidity of the bottle-brush backbone induced by the crowding and repulsion of the brush side chains^46^. The authors showed that the diameter of a superblob is equal to that of the bottle-brush cross section.

Of particular interest is the mechanical tension along the backbone of the bottle-brush polymer induced by side chain repulsion. This phenomenon occurs in solution, but its effect is particularly amplified when the bottle-brush polymer spreads on a solid substrate^39–41^. In the latter case, if the length of the bottle-brush backbone is in the range of the superblob diameter, the tension generated by side chain repulsion is focused on the middle section of the backbone, inducing its scission.

There are obvious morphological similarities between bottle-brush polymers and one-dimensional micelles formed by CDSA. The semicrystalline core can be considered as the backbone of the micelle, while the corona-forming block is equivalent to the bottle-brush side chains. The semi-crystalline core of 1D micelles is, however, more fragile than the bottle-brush backbone, and micelle fragmentation, which requires less energy than chain scission, could be induced in solution by corona chain repulsion. For example, O’Reilly and coworkers recently reported the fragmentation of PCL_50_-PDMA_180_ micelles (PCL = poly(*ε*-caprolactone), and PDMA = poly(N, N-dimethylacrylamide)) during their transfer from ethanol to water. The authors explained this phenomenon by the swelling of the PDMA corona in water that induced stress along the semicrystalline core, promoting its fracture^47^.

**Fig. 3:**
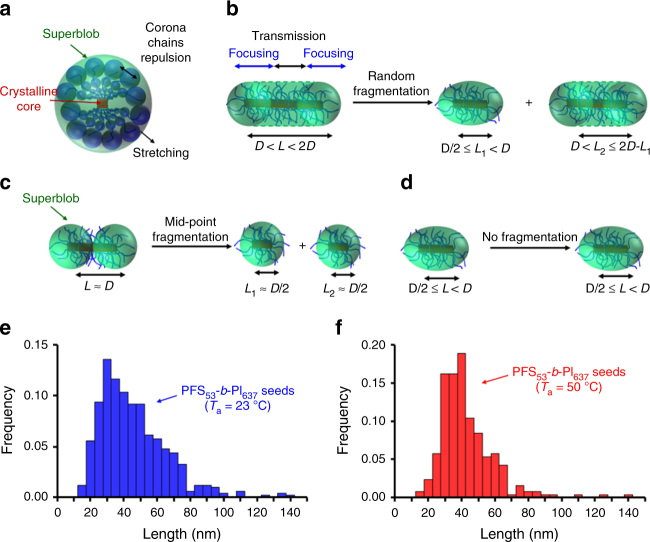
Seed fragmentation. **a** schematic representation of the cross-section of a seed crystallite. The core, a central crystalline layer sandwiched by two amorphous layers, is surrounded by the corona chains that form superblobs of diameter *D*. Schematic representations of the fragmentation of seeds of core length, *L*, due to corona chain repulsion^40^ in the case of **b** D < *L* < 2D, **c** *L* ≈ D, and **d** D/2 ≤ *L* < D. Length distribution histograms of the PFS_53_-*b*-PI_637_ seeds **e** before annealing, and **f** after annealing at 50 °C

To further deepen the analogy between core-crystalline one-dimensional micelles and bottle-brush polymers, we depict a PFS BCP micelle as a semi-crystalline core sandwiched by two amorphous layers and surrounded by a superblob (Fig. 3a). The diameter of the superblob, *D*, can thus be equated to the hydrodynamic cross-section of the PFS_53_-*b*-PI_637_ seeds, which, for a similar sample of PFS_53_-*b*-PI_637_ seeds is ca. 64 nm (see “Evaluation of the hydrodynamic diameter of PFS_53_-*b*-PI_637_ seed cross-section” in the Supplementary Discussion)^48^. If the seed crystallites were all uniform in length, with *L*_n_ = 43.5 nm, close to the value of *D*/2 (32 nm) (Supplementary Figs 15 and 16), one would not expect any fragmentation of the seeds. The length distribution of the sonicated seeds, however, is rather broad (*L*_w_/*L*_n_ = 1.22), with an average length *L*_n_ = 43.5 nm. The individual seed lengths range from 15 nm to ca. 130 nm, i.e., up to 2*D*). According to Park et al.^41^, one can separate these structures into three categories (Fig. 3b–d). The largest seeds (*D* < *L* ≤ 2*D*, Fig. 3b) would fragment randomly until they become smaller than *D*. The seeds of length *L* ≈ *D* would break in the center (Fig. 3c) and, finally, the shortest seeds (*L* < *D*) should not fracture because the tension applied to the core by the corona is not sufficient (Fig. 3d). One would thus expect the seed length distribution to become narrower, with an increase in the number of seed fragments of length *L* ≈ 32 nm. In addition, the number of seeds longer than *D* should decrease, while the population of short seed fragments (*L* < 32 nm) should not change.

To test this hypothesis, we plot, in Fig. 3e, f, the histogram of the PFS_53_-*b*-PI_637_ crystallites after sonication (Fig. 3e), and that of the seed crystallites that were annealed for 30 min at 50 °C, where fragmentation was prominent (Fig. 3f).

The length distribution of the crystallites at 23 °C (Fig. 3e) resembles a Zimm–Schulz distribution, tailing at large seed lengths. In comparison, when the seed crystallites were annealed at 50 °C (Fig. 3f), the length distribution became more symmetrical (Gaussian-like), with a noticeable decrease in the number of seeds larger than 65 nm, and an increase in the number of seeds of lengths ranging from 30 to 45 nm. This dispersion in fragment lengths leads to a number average length, *L*_critic_ ≈ 39.5 nm, larger than the expected value *D*/2 = 32 nm.

Finally, since the size of the superblob is directly related to the thickness of the corona, *D*, and the temperature dependence of the superblob size is relatively weak (*τ*^1/4^), we infer that the critical seed length would remain almost constant with temperature.

### Seed dissolution

Because polymer crystallization is a kinetic phenomenon, core-crystalline micelles do not form under thermodynamic equilibrium. This implies that the amorphous layer contains multiple polymer repeat units per fold, i.e., the value of *α* (eq. 2) is not equal to 1. In addition, the value of *α* is not constant along the micelle core (some sections of the core being more crystalline than others). Upon sonication, the fragments formed have different crystallinities. We describe this variation in micelle fragment crystallinity by a normal distribution.

To relate the distribution of dissolution temperatures to the variation in crystallinity of the seeds, we made two simple assumptions: (i) seeds with the same crystallinity dissolve at the same temperature, and (ii) the less crystalline seeds are less stable and dissolve at lower temperatures. Therefore, we expect the distribution of dissolution temperatures of the seeds to be similar to that of the distribution of crystalline seeds, i.e., to be represented by a normal distribution (Supplementary Fig. 17). The maximum value of the distribution of dissolution temperatures corresponds to the temperature at which half of the seed population has dissolved.

**Fig. 4:**
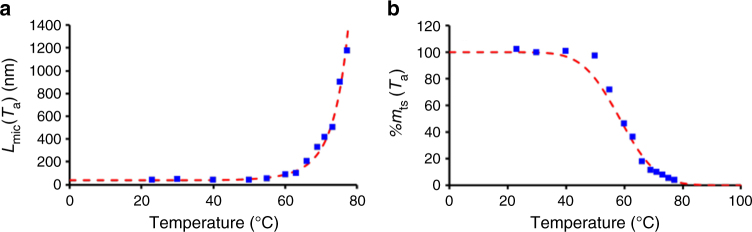
Fitting of the length of micelles obtained by self-seeding. **a** Fit (eq. 6, dashed line) of the number average length of the PFS_53_-*b*-PI_637_ seed crystallites regrown at 23 °C (filled squares) as a function of annealing temperature (control experiment). **b** Fit (dashed line) of the apparent mass percentage of surviving seeds as a function of annealing temperature (filled squares)

Above 50 °C, the distribution of trapped seed lengths does not change, which indicates that fragmentation becomes negligible, and seed dissolution predominates. Thus, at these higher temperatures, if the core crystallinity is large enough, the seed crystallites survive the tension induced by the repulsion and the stretching of the corona chains that pull on the core in all directions, while if the seed is not crystalline enough, it dissolves in a catastrophic event.

Each seed that dissolves adds a number of unimers proportional to *L*_critic_ into the solution, and the variation of *L*_mic_(*T*_*a*_) as a function of *T*_a_ (Fig. 2a) can be written (for the development of equation 6, see “Calculation of *L*_mic_(*T*_a_) as a function of *T*_a_” in the Supplementary Discussion):

6 $$L_{{\mathrm{mic}}}(T_{\mathrm{a}}) = \frac{{2L_{{\mathrm{critic}}}}}{{\mathrm{erfc}\left[ {\left( {T_{\mathrm{a}} - T_{\mathrm{d}}} \right)/\sqrt 2 \sigma } \right]}}$$

where erfc(*x*) is the complementary error function, *T*_d_ is the average dissolution temperature, and *σ* is the standard deviation.

The model developed above offers important insights into the dissolution of seed crystallites. To fit the data, we set *L*_critic_ = 40.1 nm, which corresponds to the length of the trapped seeds annealed at 50 °C. At this temperature, dissolution is minimal (less than 5% of the seeds dissolved), while fragmentation is maximum. This value is also close to the experimental average length of the trapped seeds, *L*_ts, av_ ≈ 39.5 nm. We obtained an excellent fit of *L*_mic_(*T*_a_) over the entire range of temperatures (Fig. 4a) setting *T*_d_ = 58.5 °C, and *σ* = 10 °C. This excellent fit shows that the length of the regrown micelles is only dictated by the distribution of dissolution temperatures of the seeds, and it validates the assumption that the seed dissolution temperatures follow a normal distribution (Supplementary Fig. 17).

It is often observed^32,34,35,49^ that the fraction of surviving seeds appears to decrease exponentially with increasing temperature for a carefully selected temperature range (Supplementary Figure 18). By combining eqs 1 and 6 we learn, however, that the number of surviving seeds at a given temperature should be evaluated using the distribution in crystallinity of the seed core at room temperature. This mechanism implies that the decrease in the mass percentage of surviving seeds is not strictly exponential, and arises as a direct consequence of the normal distribution in crystallinity of the seed core (Fig. 4b). This idea should not only be valid for the dissolution of polymer crystals in solution (either 1D or 2D)^50^, but also for the melting of polymer crystals in bulk^32^.

## Discussion

In summary, we have demonstrated that a seed trapping protocol can be used to investigate the dissolution of core crystalline 1D micelles. Interestingly, we observed that seed crystallites that survived dissolution were always longer than a critical length, which was independent of the temperature of annealing. This key observation allowed us to distinguish two processes that happen during annealing: fragmentation and dissolution. Using the obvious similarities between one-dimensional polymeric micelles and bottle-brush polymers, we hypothesized that the seeds will first fragment half way along the crystalline core, where the tension due to chain repulsion is maximum, to reach a critical length equal to the radius of a superblob. When the seed core is not crystalline enough, the stability of the seeds inside the superblob is compromised, and the seeds dissolve in a cooperative process that involves the simultaneous motion of multiple chains. In this regard, seed dissolution as a function of temperature can solely be related to a normal distribution in crystallinity of the core of the seeds. This idea was validated by the excellent fit of the percentage of surviving seeds versus annealing temperature. More important, this analysis goes beyond the system studied here and can be generalized to any polymer crystal.

## Methods

### Polymer synthesis

PFS_53_-*b*-PI_637_ (*M*_n, GPC_ = 56 300, Ð = 1.01) and PFS_60_-*b*-PDMS_660_ (*M*_n, GPC_ = 63 700, Ð = 1.06) were the same samples reported in ref. ^25^.

### Seed fragment preparation

The seed fragments were generated in two steps. First a mother solution of long PFS_53_-*b*-PI_637_ micelles in decane was prepared by heating ca. 5 mg of the BCP in 10 mL decane at 100 °C for 20 min. The polymer sample dissolves completely at this temperature to form very long (>10 µm) micelles upon cooling. After three days of aging at room temperature, the sample was subjected to mild sonication (4 periods of 10 min in a 50 watt sonication bath at 23 °C) to obtain short PFS_53_-*b*-PI_637_ seeds (*L*_n_ = 43.5 nm, s.e.m.  = 5%). This mother solution was then diluted to a concentration of 20 μg/mL. The same mother solution was used for all the experiments.

### Preparation of the PFS_60_-b-PDMS_600_ unimer solutions

PFS_60_-*b*-PDMS_600_ unimer solutions used to trap the surviving seeds were prepared by dissolving PFS_60_-*b*-PDMS_600_ in decane (5 mg/mL) at 100 °C. At this temperature, the sample dissolved completely. An aliquot (20 μL) of this hot solution was then injected to the seed solution, resulting in a 5-fold excess of PFS_60_-*b*-PDMS_600_ unimer over the total amount of PFS_53_-*b*-PI_637_ present in solution.

### Dissolution experiments

Twelve different annealing temperatures were investigated (RT = 23°, 30°, 40°, 50°, 55°, 60°, 63°, 66°, 69°, 71°, 73°, 75°, and 77 °C). For each temperature, two vials each containing 1 mL of seeds in decane (*c* = 20 μg/mL) were heated at the targeted annealing temperature for 25 min. Then a hot unimer solution (20 μL) of PFS_60_-*b*-PDMS_600_ in decane was added to one of the vials. The solution was slightly swirled and annealed five more minutes at the annealing temperature. Finally, both vials were removed at the same time from the heating bath and allowed to cool at room temperature. After one day, 0.1 mL of Karstedt’s catalyst was added to each solution of M_(PFS-PI)_-*r*-M_(PFS-PDMS)_-*b*-M_(PFS-PI)_-*b*-M_(PFS-PDMS)_-*r*-M_(PFS-PI)_ co-micelles. The solutions were then aged one more day at 23 °C before the co-micelles were imaged by TEM. The second series of samples (control experiments) was allowed to age for two days to ensure complete regrowth of the micelles. After aging, the regrown micelles were imaged by TEM.

### Growth kinetics study

For this experiment, we used the same mother solution of PFS_53_-*b*-PI_637_ seeds in decane as described above but after careful storage at 23 °C in a sealed vial for one year. At this temperature, the seeds were stable in solution and their length did not change (Supplementary Fig. 10). The mother solution was diluted to reach a concentration of *c* = 20 μg/mL. A vial containing 4 mL of the diluted seed solution was then annealed at 75 °C in an oil bath. We annealed the solution at 75 °C for 95, 415, 1195, and 2635 min. After each annealing time, we transferred 1 mL of the solution into an empty vial that was also pre-heated to 75 °C. Half (0.5 mL) of the solution was then injected in a second preheated empty vial, and after 5 min at 75 °C was allowed to cool to room temperature. To the second half, we added a 5-fold excess of PFS_60_-*b*-PDMS_660_ unimer that was pre-heated in decane at 100 °C. This second sample was briefly swirled, and after 5 min at 75 °C was allowed to cool to room temperature. After one day of aging at room temperature, we added 0.5 mL of decane to the trapped seed solution, followed by 0.1 mL of Karstedt’s catalyst. The trapped seeds were aged for one more day prior to be imaged by TEM. In parallel, the control samples were aged at room temperature for two days, and the regrown micelles were imaged by TEM.

### Data availability

The data that support the findings of this study are available from the corresponding authors upon reasonable request.

## Electronic supplementary material

Supplementary Information(PDF 4039 kb)
