# Supplementary material for: Explosive dissolution and trapping of block copolymer seed crystallites
Source: Nat Commun. 2018 Mar 20;9:1158. doi: 10.1038/s41467-018-03528-x (PMC5861044; doi:10.1038/s41467-018-03528-x)
Supplement: Supplementary file 1 — Supplementary Information(PDF 4039 kb) [file 41467_2018_3528_MOESM1_ESM.pdf]

Supplementary Information

Explosive Dissolution and Trapping of Block Copolymer Seed Crystallites

Guerin *et al.*

CONTENTS

Supplementary Methods	Pages 3-4
Supplementary Discussion	Pages 5-10
Supplementary Tables	Pages 11-12
Supplementary Figures	Pages 13-30
References	Page 31

SUPPLEMENTARY METHODS

Materials

Decane (99+ %) was purchased from Aldrich and used without further purification. Platinum-divinyltetramethyldisiloxane complex in xylenes (Karstedt's catalyst) with a concentration of Pt of ~2 wt % was purchased from Aldrich.

Transmission electron microscopy

Bright-field transmission electron microscopy (TEM) images were taken using a Hitachi H-7000 instrument. Samples were prepared by placing one drop of solution on a Formvar-carbon coated grid, touching the edge of the droplet with a filter paper to remove excess liquid and allowing the grid to dry. We observed that the amount of micelles deposited on a TEM grid increased when the seed solution was left for a longer contact time on the TEM grid before removing excess liquid. A sample that contained fewer seeds in the solution (after dissolution) was thus left for a longer time on the TEM grid to compensate for the decrease in number density of micelles.

For each sample, micelle length distributions were determined by tracing more than 200 micelles or stained trapped seeds using the software ImageJ (NIH, US). The number average length of the micelles (or trapped stained seeds), L_n, was calculated as

$$L_n = \frac{\sum_{i=1}^{N} N_i L_i}{\sum_{i=1}^{N} N_i} \quad (1)$$

where N_i is the number of micelles of length L_i, and N is the total number of micelles (or stained trapped seeds) examined for each sample.

Light Scattering Equipment

Static (SLS) and dynamic light scattering (DLS) measurements were performed using a wide angle light scattering photometer from ALV. The light source was a JDS Uniphase He-Ne laser (λ = 632.8 nm, 35 mW) emitting vertically polarized light. The cells were placed into the ALV/DLS/SLS-5000 Compact Goniometer System and sat in a vat of toluene, which matched

the index of refraction of the glass cells. The scattered light was detected by a Dual ALV-High Q.E. APD avalanche photodiode module, interfaced to the ALV-5000/EPP multiple tau digital. All measurements were carried out at 20 °C. SLS and DLS experiments were performed simultaneously in an unpolarized configuration, i.e., without any polarizer installed in front of the detector. The angular range consisted of scattering angles between 20º and 150º (at 5º intervals). Toluene was used as the standard in the SLS measurements.

SUPPLEMENTARY DISCUSSION

Calculation of the mass percentage of seeds that survived the annealing procedure, %$m_{ts}(T_a)$ (eq.1 main text)

When no unimer is added to a solution of micelle fragments (seed crystallites or seeds), the unimer present when the solution is heated to $T = T_a$ is due only to the dissolution of the seeds.

A seed solution annealed at a temperature T_a for a time t will contain a mass of seeds, $m_{ts}(T_a)$:

$$m_{ts}(T_a) = N_{agg/L}\ L_{ts}(T_a)\ M_0\ N_{ts}(T_a) \tag{2}$$

where $N_{agg/L}$, the linear aggregation number, describes the number of block copolymer (BCP) molecules per nanometer of seed length. $L_{ts}(T_a)$ is the number average length of the trapped seeds, M_0, the weight average molecular weight of the BCP, and $N_{ts}(T_a)$ is the number of seeds remaining in the solution at T_a. Note that no change in the magnitude of $m_{ts}(T_a)$ was observed when the sample was heated for more than 10 minutes.

The magnitude of $N_{ts}(T_a)$ depends on both the extent of seed dissolution, which decreases the number of seeds, and seed fragmentation, which causes an increase in the number of seeds. At room temperature, all of the BCP is incorporated into micelles and no unimer remains in solution. $N_{ts}(T_a)$ can thus be evaluated experimentally as a function of the number of seeds in the solution at RT prior to annealing, $N_{seeds,RT}$:

$$N_{ts}(T_a) = \frac{L_{seeds,RT}}{L_{mic}(T_a)} N_{seeds,RT} \tag{3}$$

where $L_{seeds,RT}$ is the number average length of the seeds at RT. $N_{seeds,RT}$ can also be calculated as a function of mass of seeds in solution at RT:

$$N_{seeds,RT} = \frac{m_{seeds,RT}}{N_{agg/L}\ L_{seeds,RT}\ M_0} \tag{4}$$

Replacing $N_{ts}(T_a)$ in Supplementary Equation 2 by the expressions given in Supplementary Equations 3 and 4 leads to:

$$m_{ts}(T_a) = \frac{L_{ts}(T_a)}{L_{mic}\left(T_a\right)}\ m_{seeds,RT} \tag{5}$$

Note that $N_{\mathrm{agg/L}}$ and M_0 cancel out in Supplementary Equation 5. We thus do not need to know the values of these two parameters.

Since no unimer was added to the solution, the total mass of BCP at any temperature is the same as that at RT:

$$m_{\mathrm{seeds,RT}} = m_{\mathrm{uni}}(T_{\mathrm{a}}) + m_{\mathrm{ts}}(T_{\mathrm{a}}) \tag{6}$$

The mass percentage of seeds persisting in solution after annealing at T_{a} for a time t is, thus, given by:

$$\%m_{\mathrm{s}}(T_{\mathrm{a}}) = 100 \times \frac{m_{\mathrm{ts}}(T_{\mathrm{a}})}{m_{\mathrm{uni}}(T_{\mathrm{a}}) + m_{\mathrm{ts}}(T_{\mathrm{a}})} = 100 \times \frac{m_{\mathrm{ts}}(T_{\mathrm{a}})}{m_{\mathrm{seeds,RT}}} \tag{7}$$

Rearranging Supplementary Equation 5 according to Supplementary Equation 7 gives the apparent mass percentage of seeds, $\%m_{\mathrm{ts}}(T_{\mathrm{a}})$, that survived the annealing procedure (eq. 1, main text):

$$\%m_{ts}(T_a) = \frac{L_{ts}(T_a)}{L_{mic}(T_a)} \times 100 \tag{8}$$

Evaluation of the hydrodynamic diameter of PFS_{53}-b-PI_{637} seed cross-section.

The apparent diffusion coefficient of rigid rods, D_{app}, depends on q, the wavenumber. It can be expressed as a function of the translational diffusion coefficient parallel to the rod axis, $D_{\parallel}$, that perpendicular to the rod axis, $D_{\perp}$, and the rotational diffusion coefficient, D_{r}, according to the equation given by Wilcoxon and Schurr:[1]

$$D_{\mathrm{app}} = \frac{D_{\parallel} + 2D_{\perp}}{3} + 2\left(D_{\parallel} - D_{\perp}\right)\left(\frac{1}{3} - F\left(qL\right)\right) + 2L^2 D_{\mathrm{r}} G\left(qL\right) \tag{9}$$

With:

$$F(qL)=\frac{\int_{-qL/2}^{qL/2}\int_{-qL/2}^{qL/2}\left\{\frac{\sin|x-y|}{|x-y|^{3}}-\frac{\cos|x-y|}{|x-y|^{2}}\right\}dxdy}{\int_{-qL/2}^{qL/2}\int_{-qL/2}^{qL/2}\frac{\sin|x-y|}{|x-y|}dxdy} \tag{10}$$

and

$$G(qL)=\frac{1}{(qL)^{2}}\frac{\int_{-qL/2}^{qL/2}\int_{-qL/2}^{qL/2}xy\left\{\frac{\sin|x-y|}{|x-y|^{3}}-\frac{\cos|x-y|}{|x-y|^{2}}\right\}dxdy}{\int_{-qL/2}^{qL/2}\int_{-qL/2}^{qL/2}\frac{\sin|x-y|}{|x-y|}dxdy} \tag{11}$$

The translational and rotational diffusion coefficients depend on the geometry of the rod considered. They have been evaluated for different types of rigid cylinders, *i.e.*, with flat caps, spherical caps or even for tubes by Flamik and Aragon using a boundary element method (BE).[2,3] They can be expressed as a function of the ratio of the rod length to the hydrodynamic cross-sectional diameter, $p = L/d$, and the solvent viscosity, η. For cylinders with spherical caps, the translational diffusion coefficients, $D_{\parallel}$ and $D_{\perp}$, are given by:

$$D_{\parallel}=\frac{kT}{4\pi\eta L}\left[2\ln(p)+X_{\parallel}(p)\right] \tag{12a}$$

$$D_{\perp}=\frac{kT}{4\pi\eta L}\left[\ln(p)+X_{\perp}(p)\right] \tag{12b}$$

Where T is the temperature, k is the Boltzmann constant, while $X_{\perp}$ and $X_{\parallel}$, are the translational diffusion shape functions:

$$X_{\parallel}=-0.113192-\frac{1.30429}{p^{0.25}}+\frac{1.19032}{p^{0.5}}+\frac{3.12756}{p}-\frac{1.56699}{p^{2}}-\frac{0.930791\ln(p)}{p^{2}} \tag{13a}$$

$$X_{\perp}=0.902952-\frac{0.311044}{p^{0.25}}+\frac{0.18085}{p^{0.5}}+\frac{0.904672}{p}-\frac{0.344085}{p^{2}}-\frac{0.190732\ln(p)}{p^{2}} \tag{13b}$$

The rotational diffusion coefficient D_{r} is given by

$$D_r = \frac{3kT}{\pi\eta L^3}\left[\ln(p) + X_r(p)\right] \quad (14)$$

With the rotational diffusion shape, X_r, given by:

$$X_r = -0.372093 - \frac{0.95622}{p^{0.25}} + \frac{1.24792}{p^{0.5}} + \frac{1.23085}{p} - \frac{1.99498}{p^2} + \frac{1.84201}{p^3} - \frac{0.664147}{p^4} \quad (15)$$

D_{app} can also be obtained experimentally from the ratio Γ_1/q^2, where Γ_1 is the first cumulant calculated for each angle from the fitting of the autocorrelation decay with a cumulant expansion to second order (2-CUM).

To evaluate the hydrodynamic diameter of the seed cross-section, we first prepared a relatively concentrated solution (c = 6.0 mg mL^{-1}) of PFS_{53}-b-PI_{637} micelles in decane, by heating the polymer in decane for 30 min at 100 ^{o}C, and letting the solution cool to room temperature in air. We then sonicated the solution for three 10 min intervals to obtain short seeds. The solution was finally diluted to different concentrations (namely 0.05, 0.18, 0.3, 0.6, and 1 mg mL^{-1}) and multiangle static (SLS) and dynamic (DLS) light scattering experiments were performed on each sample. The seed length, L, was calculated from the averaged Guinier plot (Supplementary Figure 15), while d was used as an adjustable parameter to fit the averaged plot of D_{app} as a function of qL (Supplementary Figure 16).

Calculation of $L_{mic}(T_a)$ as a function of T_a (eq.6 main text)

In seeded growth experiments, one adds a given mass of unimer to a solution of seeds to extend their length. In self-seeding experiments, the unimer formed upon heating a micelle fragment solution comes from the dissolution of existing seeds, and, upon cooling to room temperature, this unimer adds to the seeds that survived the annealing step. There is thus a simple relationship between the length of the seeds regrown at room temperature, $L_{mic}(T_a)$, and the mass of unimer obtained by the dissolution of some of the seeds:[4]

$$L_{mic}(T_a) = L_{ts}(T_a)\left[1 + \frac{m_{uni}(T_a)}{m_{ts}(T_a)}\right] \quad (16)$$

The mass of surviving seeds, $m_{ts}(T_a)$, is given by Supplementary Equation 2, while the mass of unimer formed at T_a, is given by:

$$m_{\text{uni}}\left(T_{\text{a}}\right)=m_{\text{seeds,RT}}-m_{\text{ts}}\left(T_{\text{a}}\right) \tag{17}$$

Supplementary Equation 16 thus becomes:

$$L_{\text{mic}}(T_{\text{a}})=L_{\text{ts}}(T_{\text{a}})\left[1+\frac{m_{\text{seeds,RT}}-m_{\text{ts}}(T_{\text{a}})}{m_{\text{ts}}(T_{\text{a}})}\right]=L_{\text{ts}}(T_{\text{a}})\,\frac{m_{\text{seeds,RT}}}{m_{\text{ts}}(T_{\text{a}})} \tag{18}$$

Incorporating Supplementary Equation 2 into Supplementary Equation 18 leads to:

$$L_{\text{mic}}(T_{\text{a}})=L_{\text{ts}}(T_{\text{a}})\,\frac{m_{\text{seeds,RT}}}{N_{\text{agg/L}}\;L_{\text{ts}}(T_{\text{a}})\,M_0\;N_{\text{ts}}(T_{\text{a}})} \tag{19}$$

If we assume that the distribution of dissolution temperatures of the seeds, $f(T_{\text{a}})$, can be represented by a normal distribution (Supplementary Figure 17), then

$$f(T_{\text{a}})=\frac{\exp\left[-\left(T_{\text{a}}-T_{\text{d}}\right)^2/2\sigma^2\right]}{\sqrt{2\pi\sigma^2}} \tag{20}$$

where T_{d} is the average dissolution temperature, and σ is the standard deviation of the distribution.

As depicted in Supplementary Figure 17, the number of seeds dissolved at a given temperature is equal to the sum of all the seeds that dissolved below this temperature. The number of seeds that have survived at T_{a} is thus given by:

$$N_{\text{ts}}(T_{\text{a}})=N_0\left(1-\int_0^{T_{\text{a}}}\frac{\exp\left[-\left(x-T_{\text{d}}\right)^2/2\sigma^2\right]}{\sqrt{2\pi\sigma^2}}dx\right) \tag{21}$$

where N_0 is the total number of seeds in solution before dissolution.

Supplementary Equation 21 can be rewritten using the complementary error function, *erfc*(x):

$$N_{ts}(T_a)=N_0\,\frac{erfc\left[\left(T_a-T_d\right)/\sqrt{2}\sigma\right]}{2} \tag{22}$$

At mild temperatures (for $T_{\text{a}} \leq 50$ °C), the seeds do not dissolve to a significant extent, but they undergo a small extent of fragmentation, increasing the total number of seeds present in solution. From Supplementary Tables I and II, Supplementary Figure 3a, and Figure 2b (main text), we note that at 50 °C, less than 5% of the seeds have dissolved, and the average length of

the seeds trapped at this temperature is equal to the critical length, L_{critic}, of the surviving seeds for samples annealed at higher temperatures. We can thus assume that N_0 = $N_{ts}(50°)$, and Supplementary Equation 22 becomes:

$$N_{ts}(T_a) = N_{ts}(50°)\frac{erfc\left[\left(T_a - T_d\right)/\sqrt{2}\sigma\right]}{2} \tag{23}$$

At 50 °C, the seeds did not dissolve, we can thus write:

$$m_{seeds,RT} = m_{ts}(50°) = N_{ts}(50°)L_{critic}\ M_0 N_{agg/L} \tag{24}$$

Incorporating Supplementary Equations 23 and 24 into Supplementary Equation 19 allows us to relate the length of the micelles regrown at room temperature, $L_{mic}(T_a)$, to the distribution in temperatures of dissolution of the seeds, Equation 6 main text:

$$L_{mic}(T_a) = \frac{2L_{critic}}{erfc\left[\left(T_a - T_d\right)/\sqrt{2}\sigma\right]} \tag{25}$$

Finally, the combination of Supplementary Equations 8, and 25, with $L_{ts}(T_a)$ = L_{critic} leads to:

$$\%m_{ts}(T_a) = \frac{erfc\left[\left(T_a - T_d\right)/\sqrt{2}\sigma\right]}{2} \times 100 \tag{26}$$

SUPPLEMENTARY TABLES

Supplementary Table 1: Values of $L_{mic}(T_a)$, and of the mass percentage of the surviving seed crystallites ($\%m_{ts}(T_a)$) of PFS$_{53}$-*b*-PI$_{637}$ heated for 30 min at a given annealing temperature, T_a, and cooled to room temperature (control experiments).

T_a (°C)	$L_{mic}(T_a)$ (nm)	error [a] (%)	$\%m_{ts}(T_a)$ (%)
23	43.5	5	102
30	45.2	8	100
40	42.4	7	101
50	41.2	7	97
55	50.9	6	72
60	83.2	3	46
63	102	5	36
66	205	3	18
69	328	4	11
71	415	3	10
73	558	3	7
75	902	2	5
77	1173	3	4

a. standard error of the mean determined by tracing more than 200 micelles for each annealing temperature.

Supplementary Table 2: Values of $L_{ts}(T_a)$, of the surviving PFS_{53}-*b*-PI_{637} crystallites heated for 30 min at a given annealing temperature, T_a (seed trapping experiments).

T_a (°C)	$L_{ts}(T_a)$ (nm)	error [a] (%)
23	44.4	6.5
30	45.2	7.4
40	42.8	8.8
50	40.1	10.3
55	36.6	7.5
60	40.9	7.5
63	36.6	6.1
66	36.6	7.0
69	36.8	9.3
71	39.8	9.2
73	38.5	7.3
75	46.3	6.9
77	44.4	10.2

a. standard error of the mean determined by tracing more than 200 stained trapped seeds for each annealing temperature.

SUPPLEMENTARY FIGURES

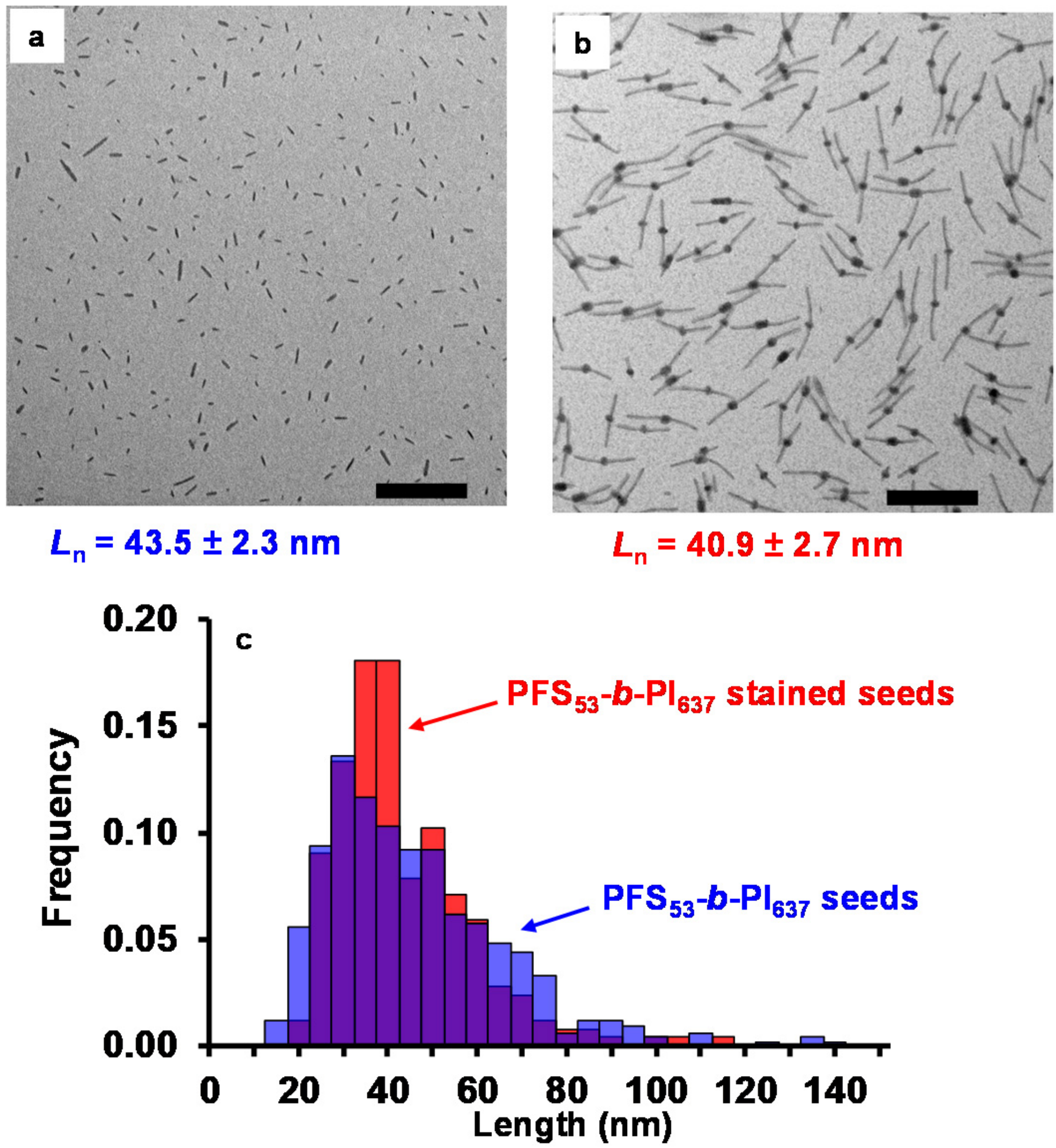

Supplementary Figure 1: TEM micrograph of a) PFS_{53}-*b*-PI_{637} seeds, b) triblock comicelles consisting of PFS_{53}-*b*-PI_{637} seeds trapped with PFS_{60}-*b*-$PDMS_{660}$ and stained with Karstedt's catalyst to highlight the PI rich section of each micelle. The PFS_{60}-*b*-$PDMS_{660}$ added in the trapping step formed the long unstained blocks at the ends of the seed fragments. c) Histograms of the lengths of the PFS_{53}-*b*-PI_{637} seeds before addition of PFS_{60}-*b*-$PDMS_{660}$ unimer (blue bars) and of the stained PFS_{53}-*b*-PI_{637} seeds after addition of PFS_{60}-*b*-$PDMS_{660}$ unimer (red bars). Scale bars, 500 nm.

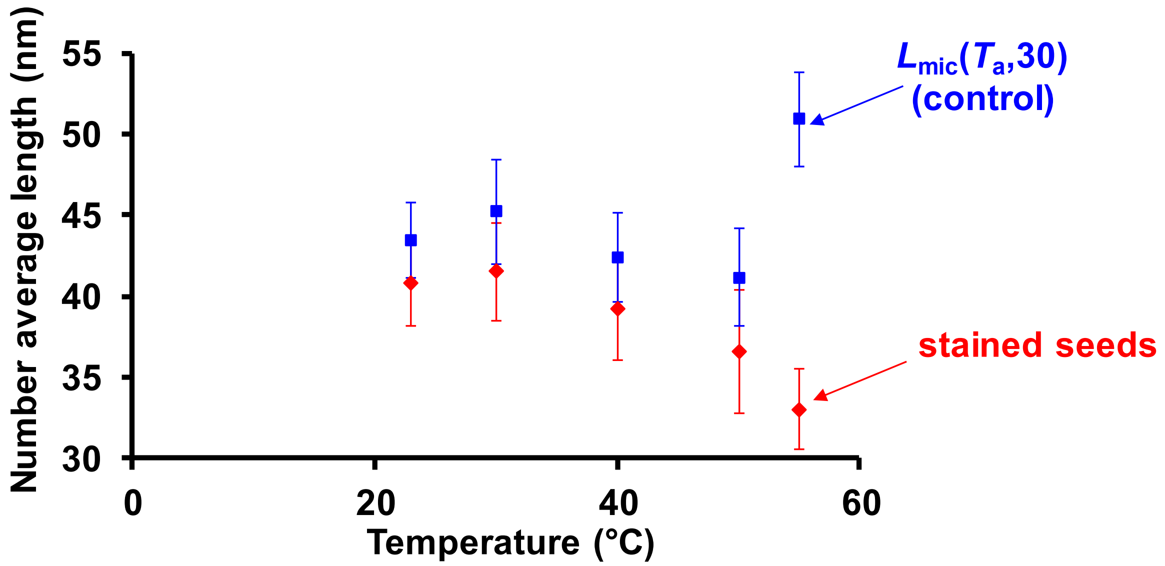

Supplementary Figure 2: a) Comparison of the lengths of the (surviving) seeds trapped after being annealed 30 min in decane at the temperature indicated. The blue points represent the number average lengths of the seeds ($L_{mic}(T_a)$) measured from the control experiment in which the seed solutions were heated, and then cooled back to RT. The red points were obtained from corresponding experiments in which the seeds were trapped with excess PFS_{60}-*b*-$PDMS_{660}$, cooled to RT, and then stained with Karsted's catalyst. The vertical lines represent the s.e.m. of the length distributions of the stained trapped seeds or of the micelles. The systematic difference of ca. 3.5 nm between the values of the control samples and the stained trapped seeds, below 50 °C, indicates that the stained PI corona chains collapsed during staining leading to an underestimation of the length of the trapped seeds by 3.5 nm. The number average length of the seeds as well as the values of $L_{mic}(T_a)$, and s.e.m. were determined by tracing more than 200 stained trapped seeds or micelles for each annealing temperature.

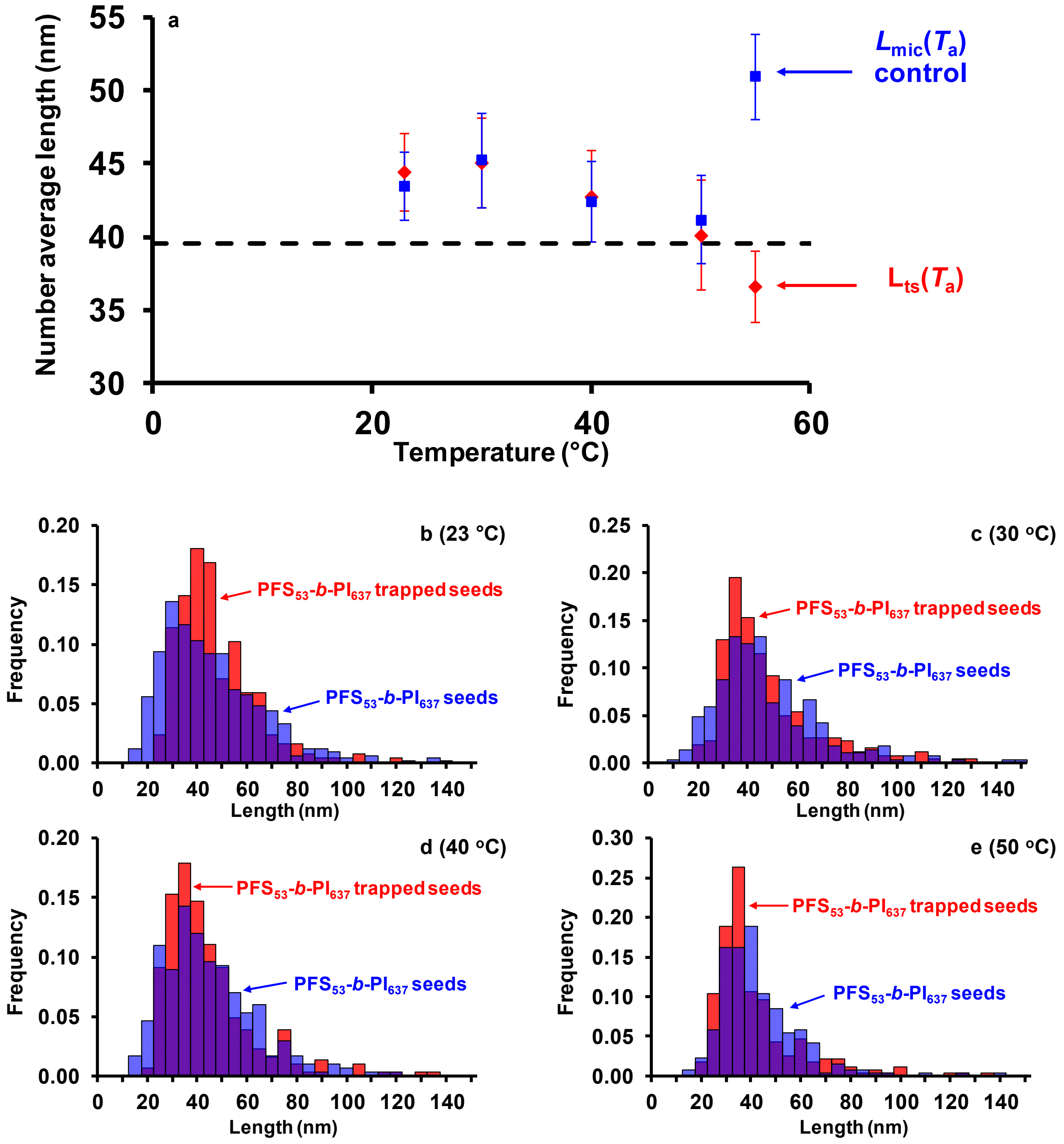

Supplementary Figure 3: a) Comparison of the lengths of the (surviving) seeds trapped after being annealed 30 min in decane at the temperature indicated. The blue points represent the number average lengths of the seeds, $L_{mic}(T_a)$, measured from the control experiment in which the seed solutions were heated and then cooled back to RT. The red points correspond to the trapped seed lengths, $L_{ts}(Ta)$, obtained by adding 3.5 nm to the lengths of the stained seeds. The vertical lines represent the s.e.m. of the length distributions. Histograms of the seed length distributions for the control experiment (blue bars) compared to that of the corresponding trapped seed length distribution (red bars) annealed 30 min at b) 23 °C, c) 30 °C, d) 40 °C, and e) 50 °C. $L_{mic}(T_a)$, $L_{ts}(T_a)$ and s.e.m. were determined by tracing more than 200 stained trapped seeds or micelles for each annealing temperature.

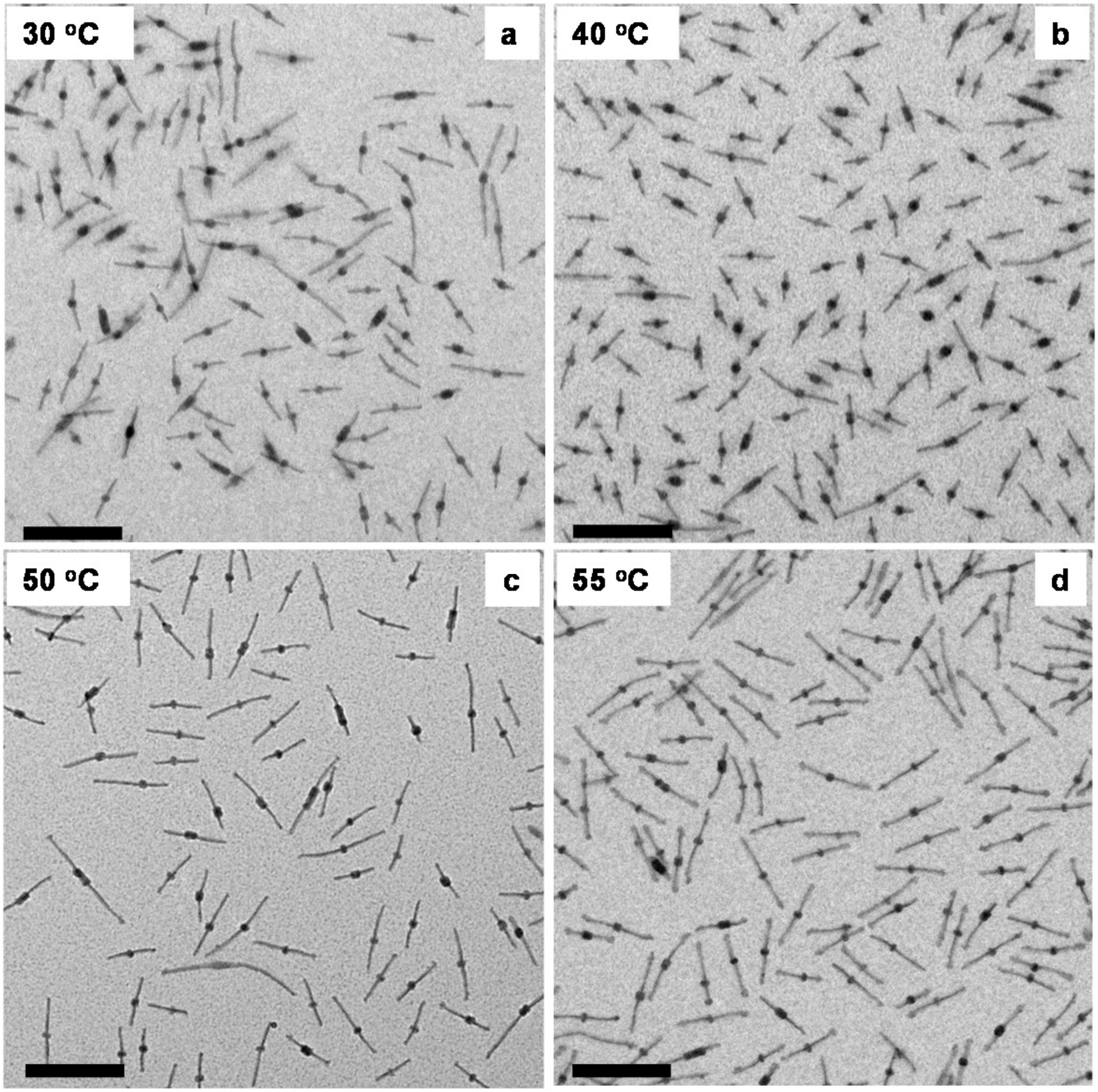

Supplementary Figure 4: TEM micrographs of trapped seeds obtained by heating solutions of PFS_{53}-*b*-PI_{637} seeds in decane for 25 min at a) 30 °C, b) 40 °C, c) 50 °C and d) 55 °C, then adding PFS_{60}-*b*-$PDMS_{660}$ unimer. The solutions were then aged for 5 more min and allowed to cool to 23 °C. The TEM images were chosen to show as many micelles as possible in each image. Note that every micelle contains a trapped seed of measurable length near the center of the micelle, demonstrating that no spontaneous nucleation of micelles occurred during the addition of PFS_{60}-*b*-$PDMS_{660}$ unimer and subsequent cooling of the samples. Scale bars, 500 nm.

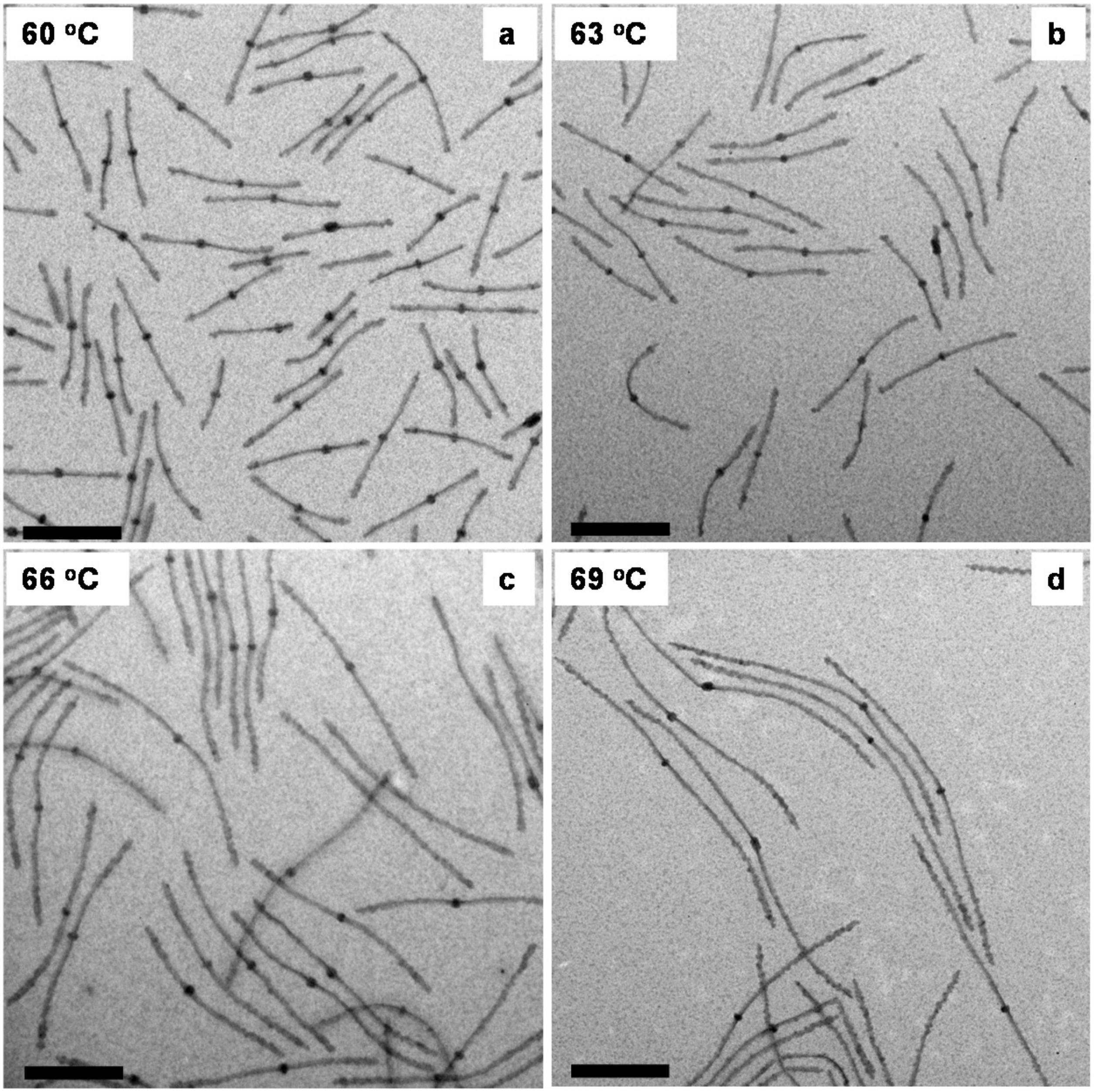

Supplementary Figure 5: TEM micrographs of trapped seeds obtained by heating solutions of PFS_{53}-*b*-PI_{637} seeds in decane for 25 min at a) 60 °C, b) 63 °C, c) 66 °C and d) 69 °C, then adding PFS_{60}-*b*-$PDMS_{660}$ unimer. The solutions were then aged for 5 more min and allowed to cool to 23 °C. The TEM images were chosen to show as many micelles as possible in each image. Note that every micelle contains a trapped seed. Scale bars, 500 nm.

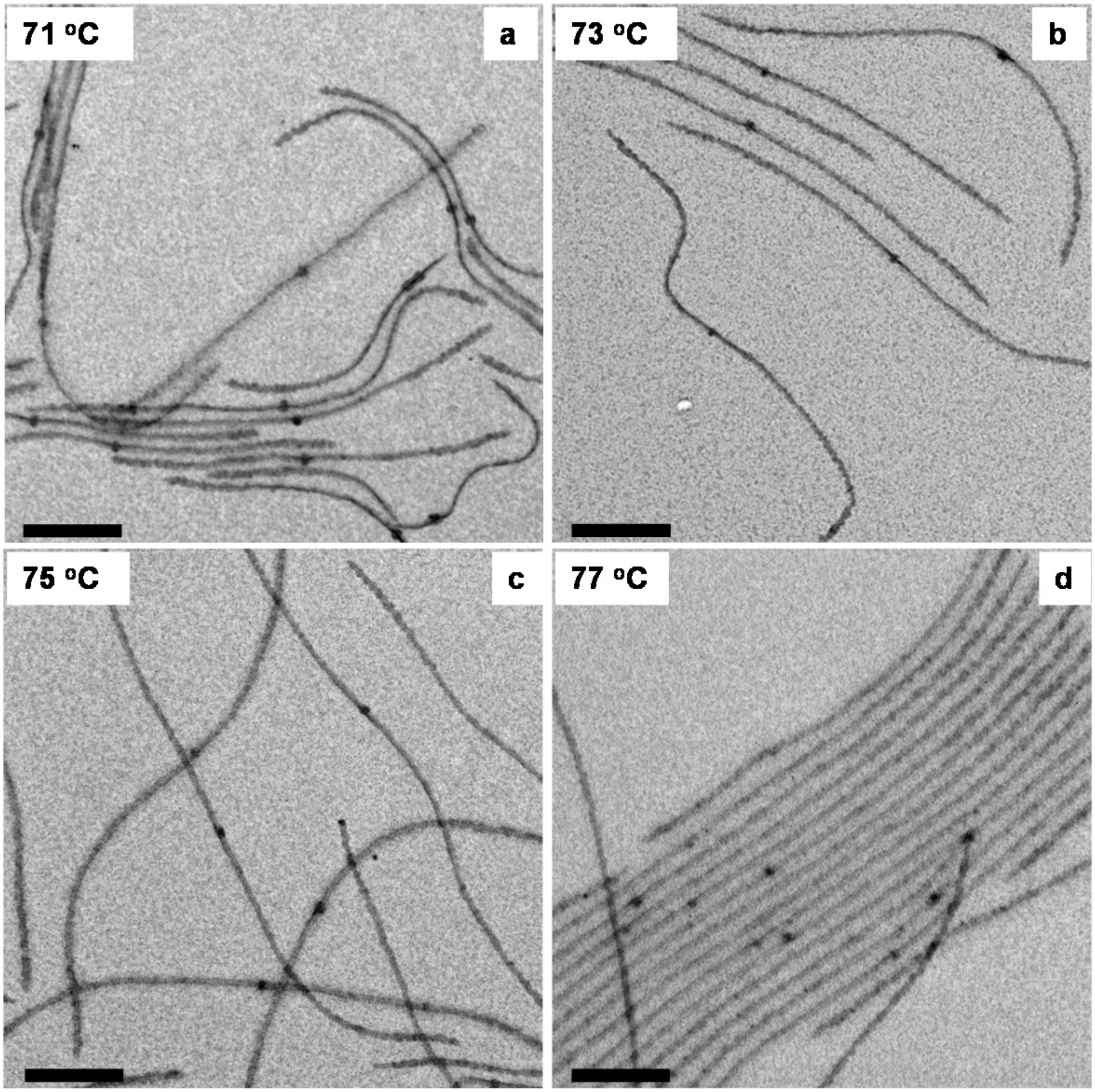

Supplementary Figure 6: TEM micrographs of trapped seeds obtained by heating solutions of PFS_{53}-*b*-PI_{637} seeds in decane for 25 min at a) 71 °C, b) 73 °C, c) 75 °C and d) 77 °C, then adding PFS_{60}-*b*-$PDMS_{660}$ unimer. The solutions were then aged for 5 more min and allowed to cool to 23 °C. The TEM images were chosen to show as many trapped seeds as possible in each image. Lower magnification images show that every micelle contains a trapped seed. Scale bars, 500 nm.

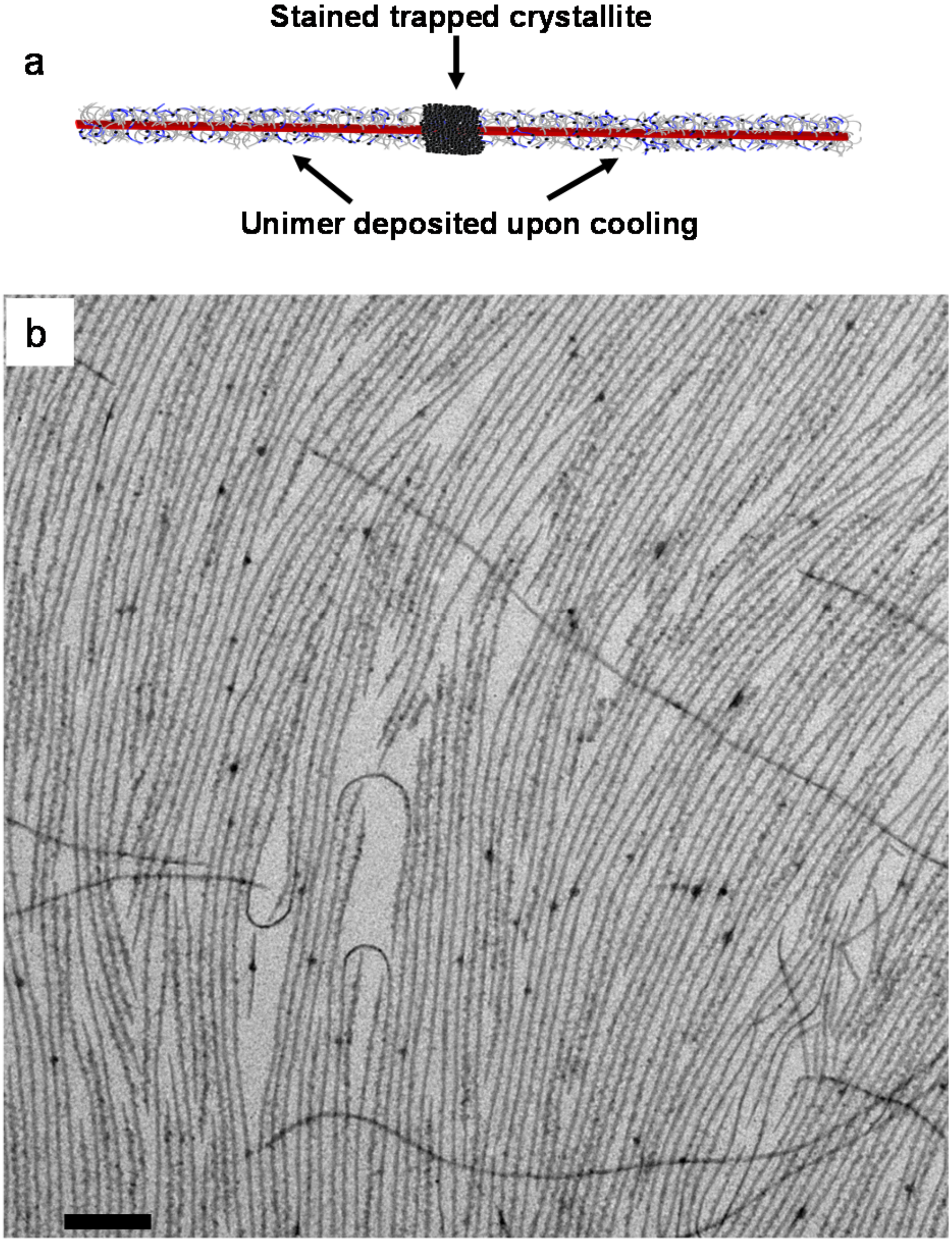

Supplementary Figure 7: a) Schematic representation of a trapped crystallite that was stained after the solution was cooled to 23 °C, and then deposited on a TEM grid. b) Low magnification TEM micrograph of trapped seeds obtained by heating a solution of PFS_{53}-*b*-PI_{637} seeds in decane for 25 min at 77 °C, then adding PFS_{60}-*b*-$PDMS_{660}$ unimer. The solution was then aged for 5 more min and allowed to cool to 23 °C. Scale bar, 500 nm.

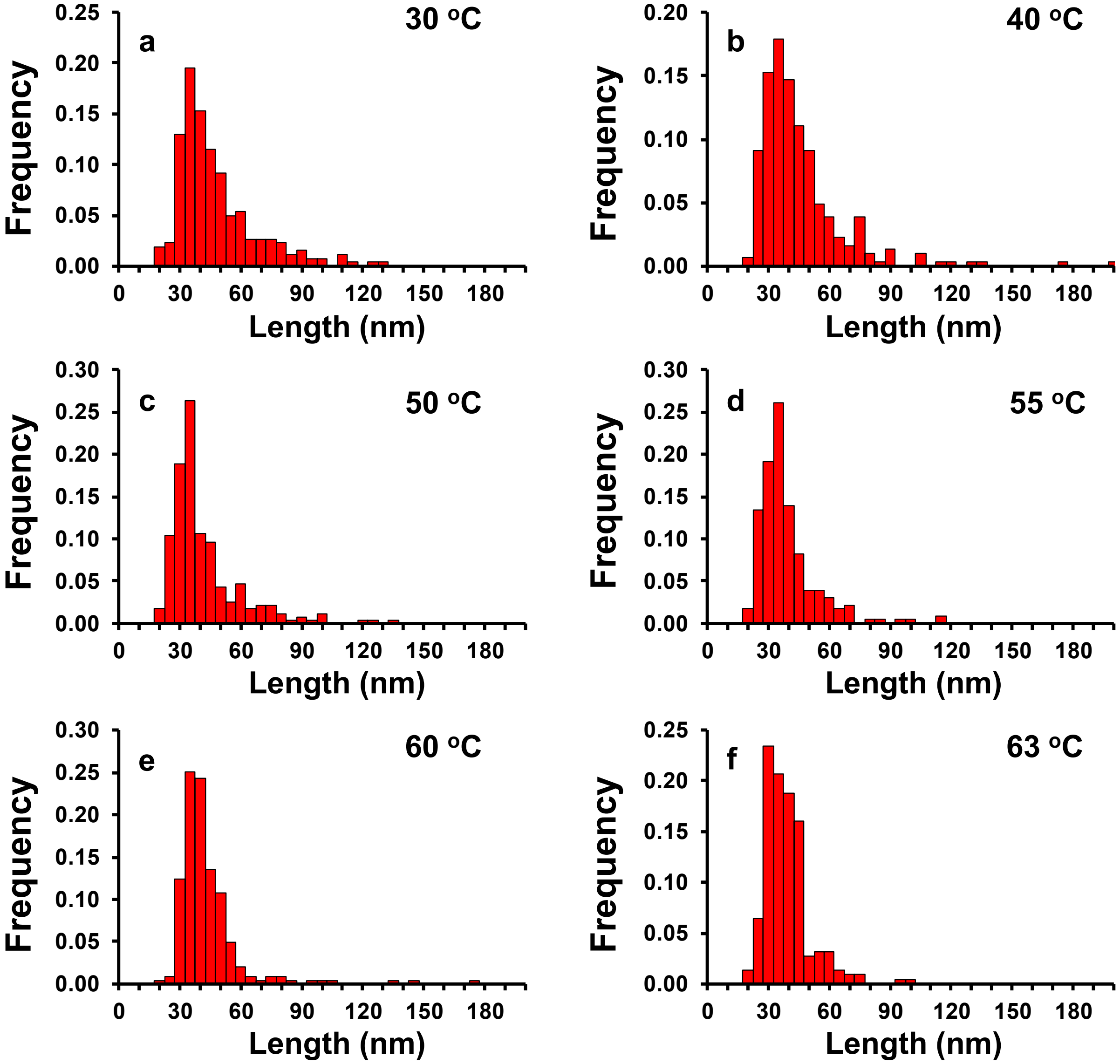

Supplementary Figure 8: Histograms of the length distributions of PFS_{53}-*b*-PI_{637} trapped seeds after the samples were annealed 30 min at a) 30 °C, b) 40 °C, c) 50 °C, d) 55 °C, e) 60 °C and f) 63 °C, and then cooled to 23 °C.

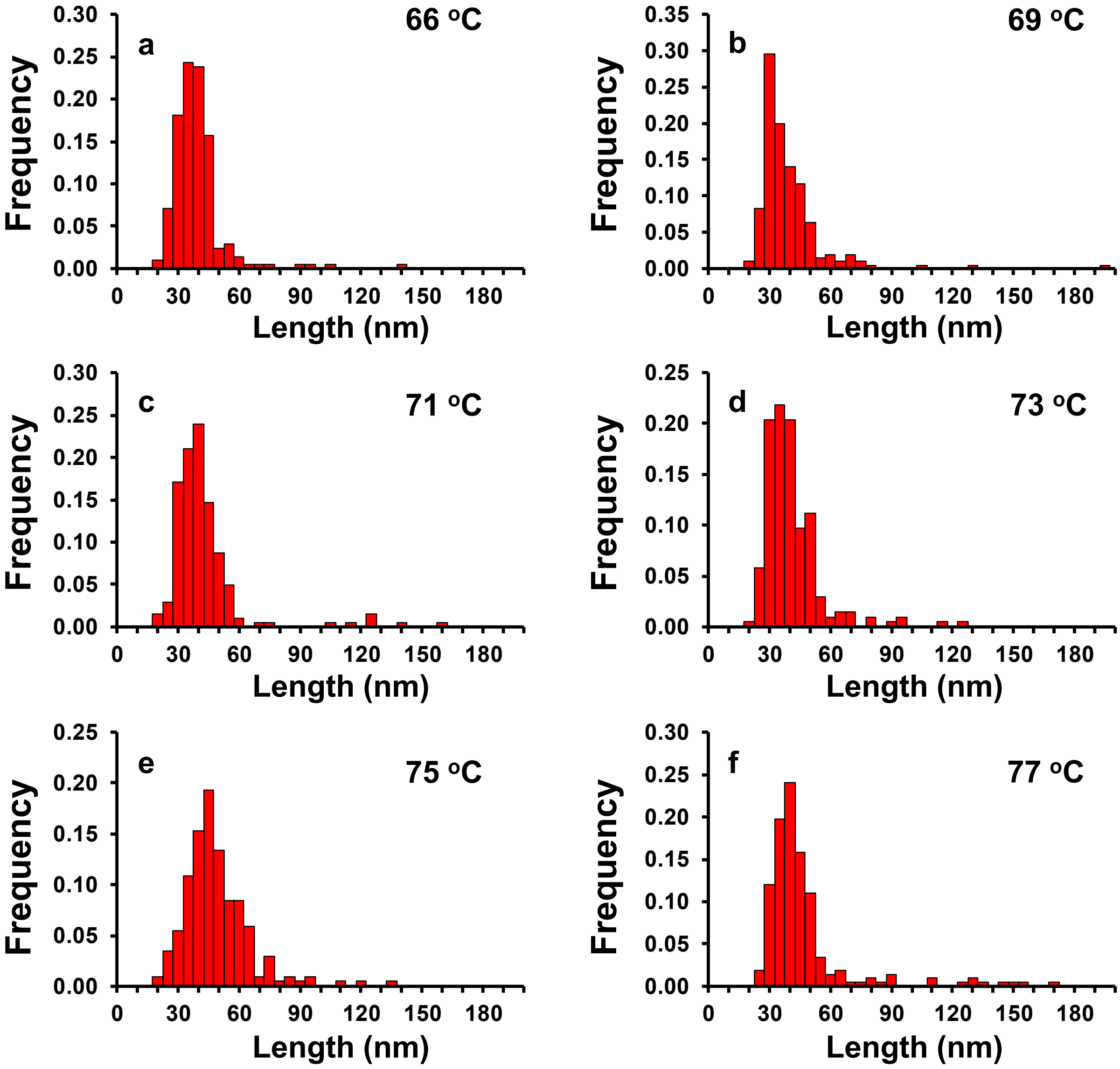

Supplementary Figure 9: Histograms of the length distributions of PFS_{53}-*b*-PI_{637} trapped seeds after the samples were annealed 30 min at a) 66 °C, b) 69 °C, c) 71 °C, d) 73 °C, e) 75 °C and f) 77 °C, and then cooled to 23 °C.

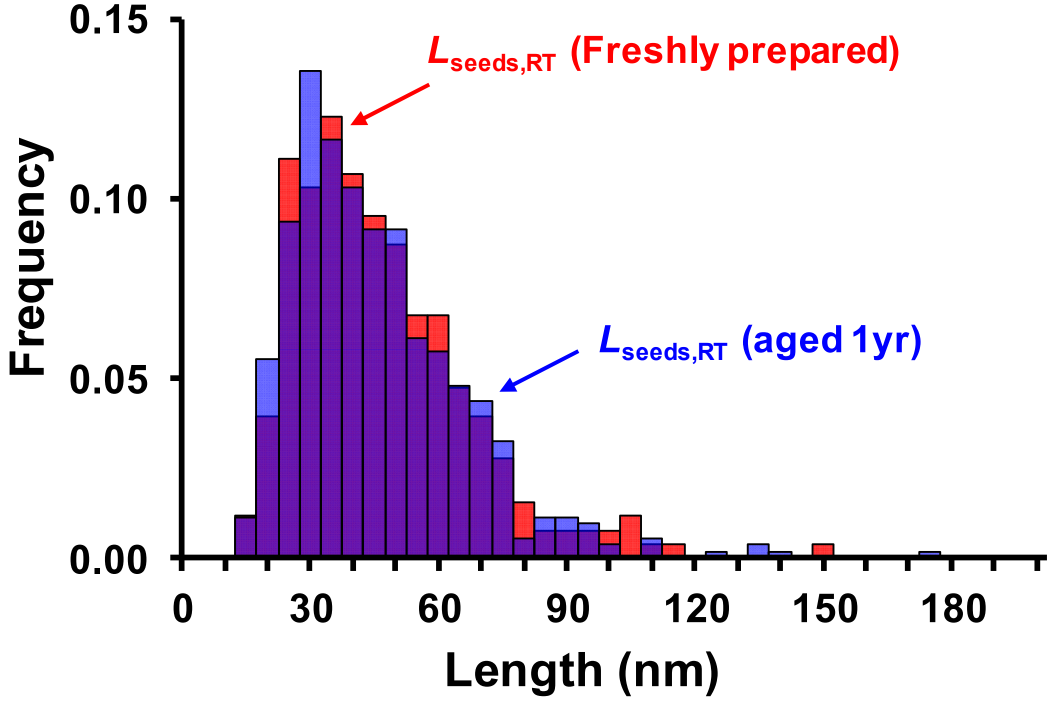

Supplementary Figure 10: Effect of PFS_{53}-*b*-PI_{637} seed crystallite history on their stability at RT. Red histogram, a solution in decane of freshly prepared PFS_{53}-*b*-PI_{637} seeds. Blue histogram, a sample of the same seed solution that was allowed to age for 1 yr at room temperature in a sealed vial. The number average length of the freshly prepared seeds crystallites was $L_{seeds,RT}$ = 43.5 nm, similar to that of the sample aged for 1 yr ($L_{seeds,RT}$ = 44.3 nm).

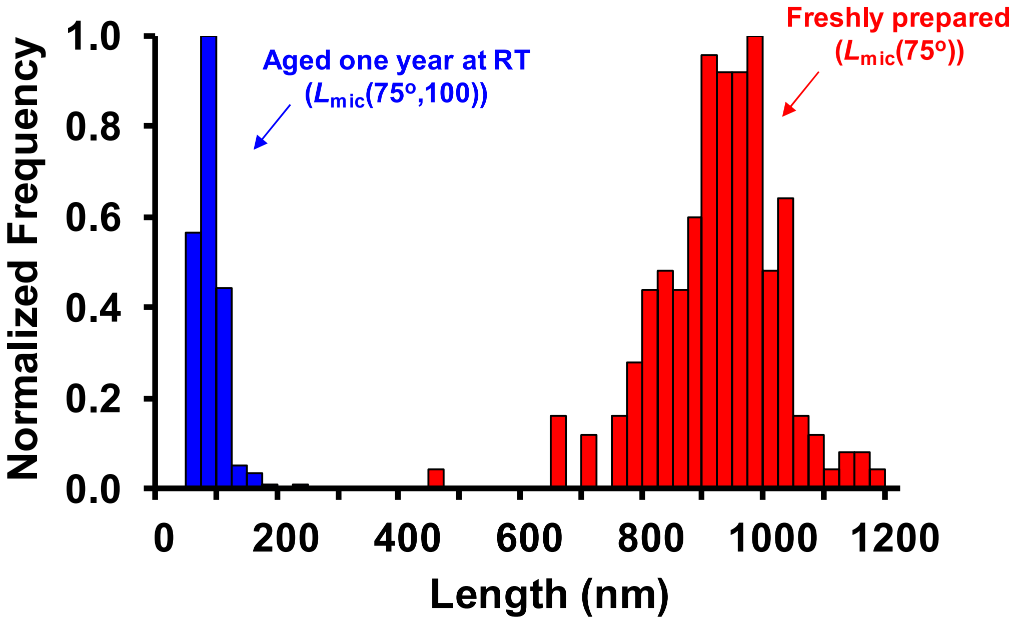

Supplementary Figure 11: Effect of PFS_{53}-*b*-PI_{637} seed crystallite history on their stability against dissolution upon heating. Red histogram, a solution in decane of freshly prepared PFS_{53}-*b*-PI_{637} seeds heated for 30 min at 75 °C and cooled to RT. Blue histogram, a sample of the same seed solution that was allowed to age for 1 yr at room temperature in a sealed vial. This solution was then heated for 100 min at 75 °C and cooled to 23 °C. The number average length of the seeds regrown from the solution aged for 1 yr was much smaller (L_{mic}(75°,100) = 64 nm) than that of the freshly prepared seeds (L_{mic}(75°) = 902 nm). This difference indicates that a much smaller fraction of the aged seeds dissolved upon heating to 75 °C, consistent with a substantial enhancement of the robustness of the seeds due to an increase in crystallinity.

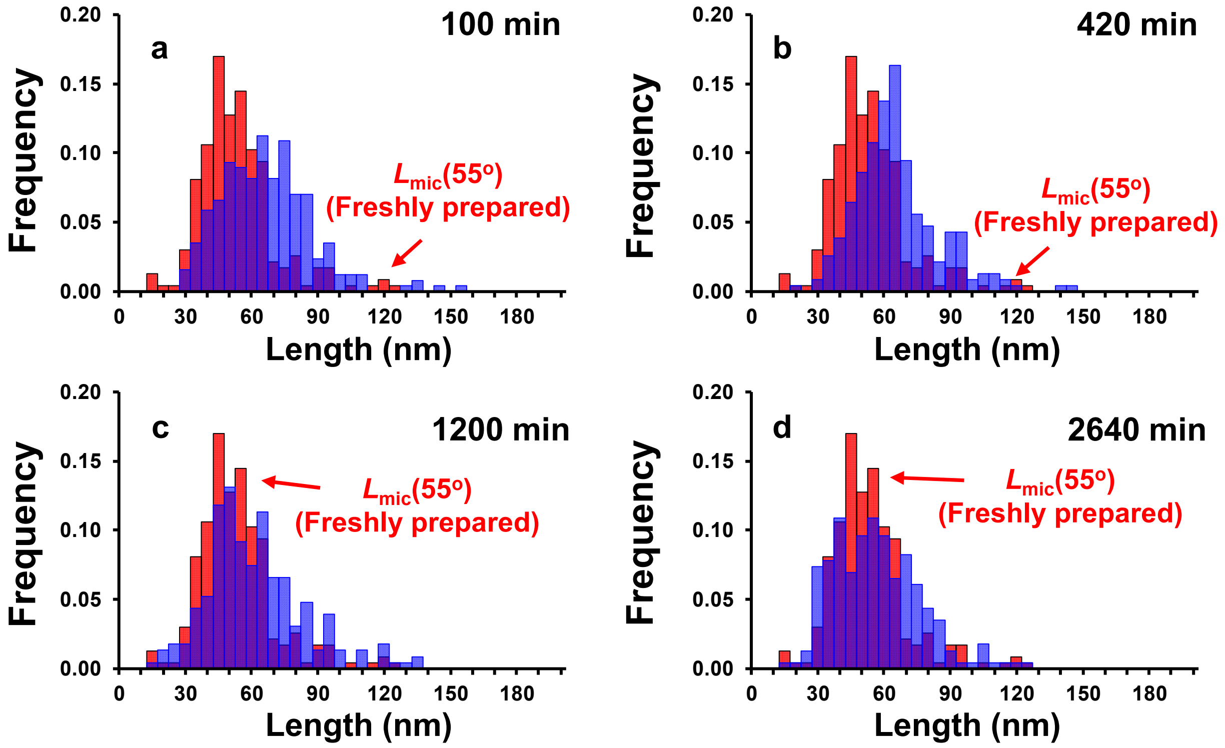

Supplementary Figure 12: Effect of PFS_{53}-*b*-PI_{637} seed crystallite history on their stability against dissolution upon heating. Histograms of the length distributions of PFS_{53}-*b*-PI_{637} seeds after a) 100 min, b) 420 min, c) 1200 min and d) 2640 min of annealing at 75 °C. The seed solution was allowed to age for 1 yr at room temperature in a sealed vial. This solution was then heated for the different annealing times indicated and cooled to 23 °C. For comparison we show the histogram obtained from a solution in decane of freshly prepared PFS_{53}-*b*-PI_{637} seeds heated for 30 min at 55 °C and cooled to RT (Red histograms).

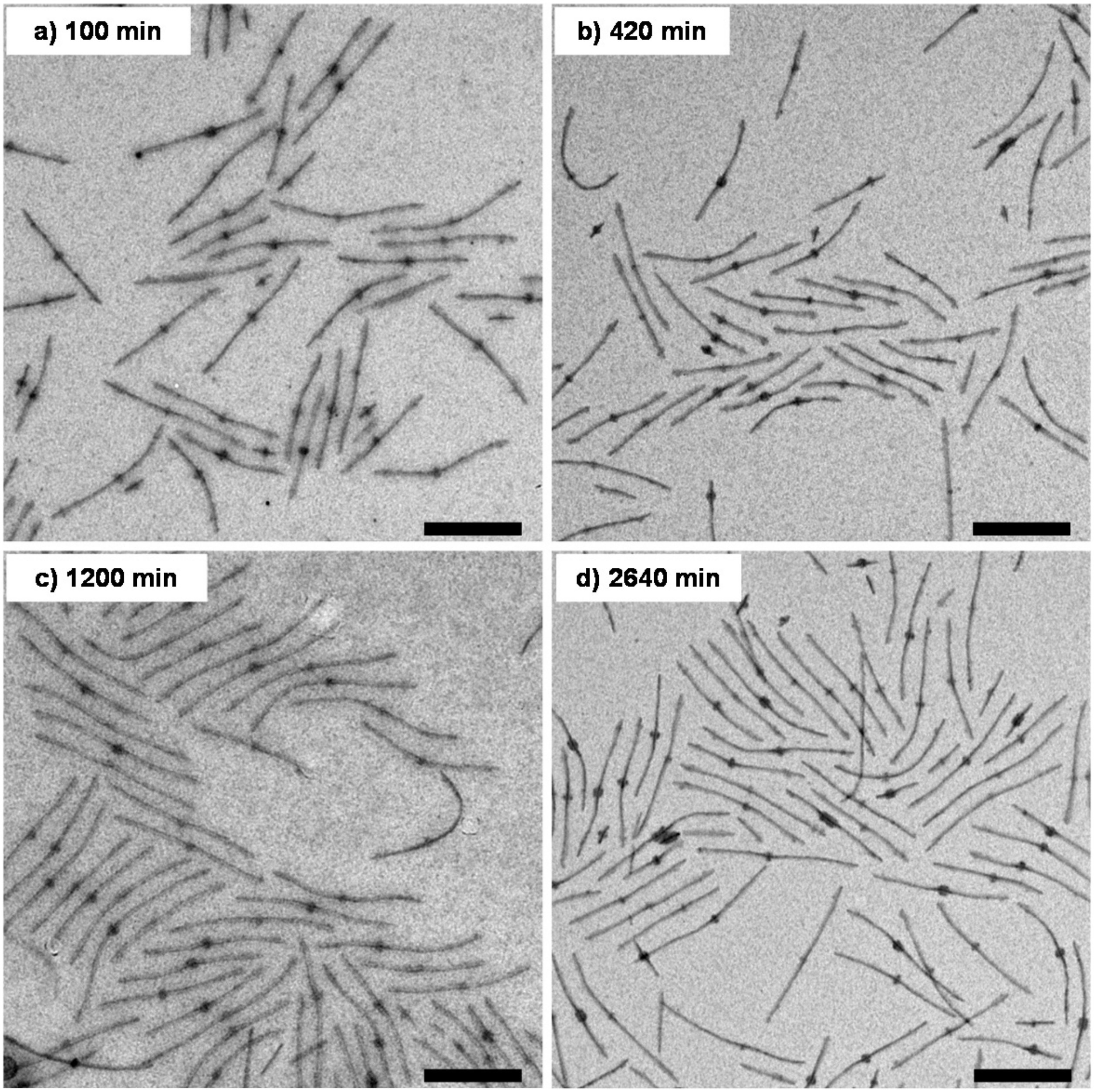

Supplementary Figure 13: TEM micrographs of PFS_{53}-*b*-PI_{637} seeds trapped after a) 100 min, b) 420 min, c) 1200 min and d) 2640 min of annealing at 75 °C. then adding PFS_{60}-*b*-$PDMS_{660}$ unimer. The solutions were then aged for 5 more min and allowed to cool to 23 °C. The TEM images were chosen to show as many trapped seeds as possible in each image. Scale bars, 500 nm.

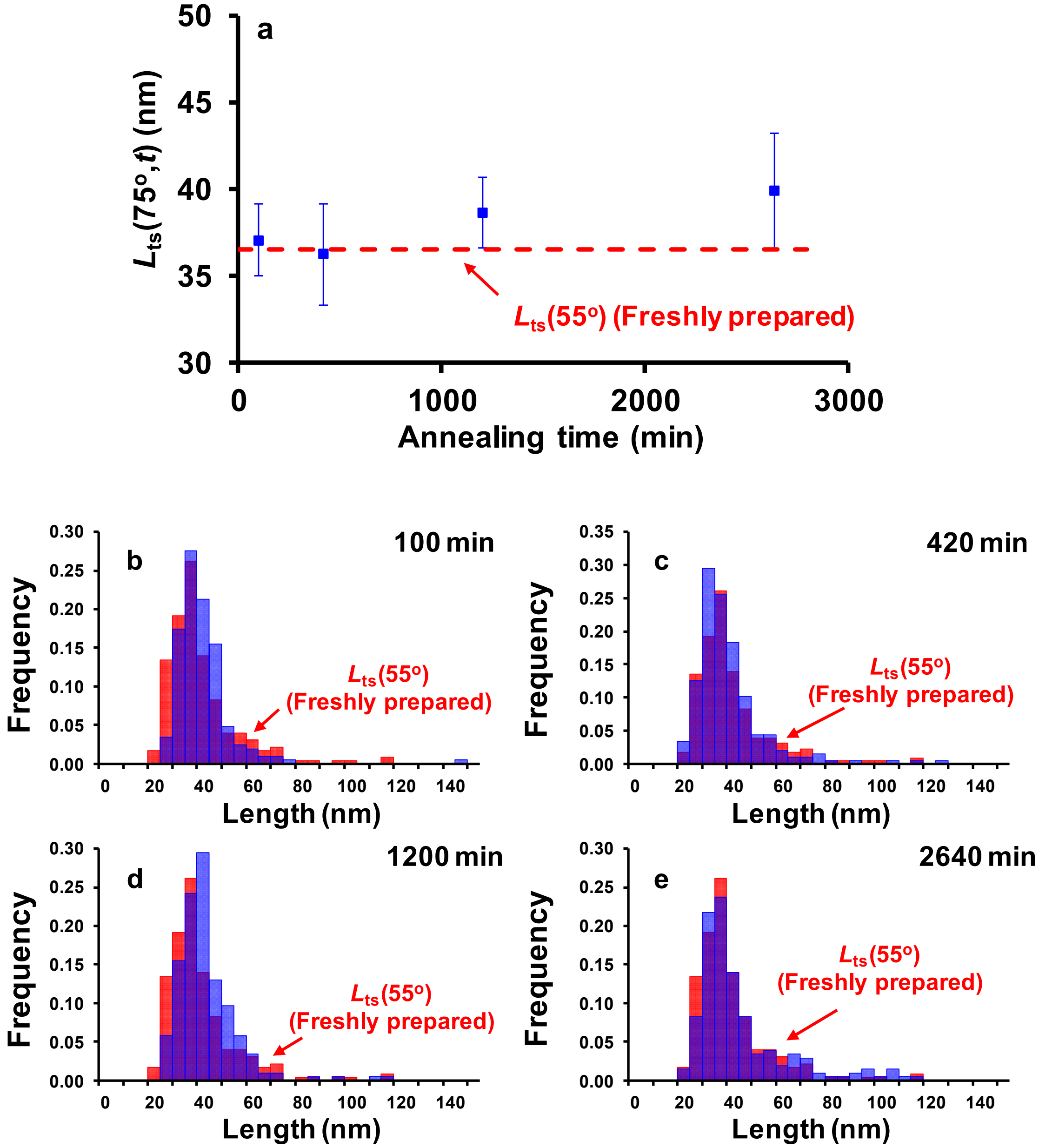

Supplementary Figure 14: Effect of PFS$_{53}$-*b*-PI$_{637}$ seed crystallite history on the critical length. a) Plot of number average length, $L_{ts}(75°,t_a)$, of seeds trapped with an excess of PFS$_{60}$-*b*-PDMS$_{660}$ after different annealing times, t_a, at 75 °C, as a function of t_a. The seed solution was allowed to age for 1 yr at room temperature in a sealed vial before the seed trapping experiment. Histograms of the length distributions of PFS$_{53}$-*b*-PI$_{637}$ trapped seeds after b) 100 min, c) 420 min, d) 1200 min and e) 2640 min of annealing at 75 °C. For comparison we show the histogram obtained from a solution in decane of freshly prepared PFS$_{53}$-*b*-PI$_{637}$ seeds heated for 30 min at 55 °C, and trapped with an excess of PFS$_{60}$-*b*-PDMS$_{660}$ (Red histograms). The values of $L_{ts}(75°, t_a)$ and s.e.m. were determined by tracing more than 200 stained trapped seeds for each annealing time.

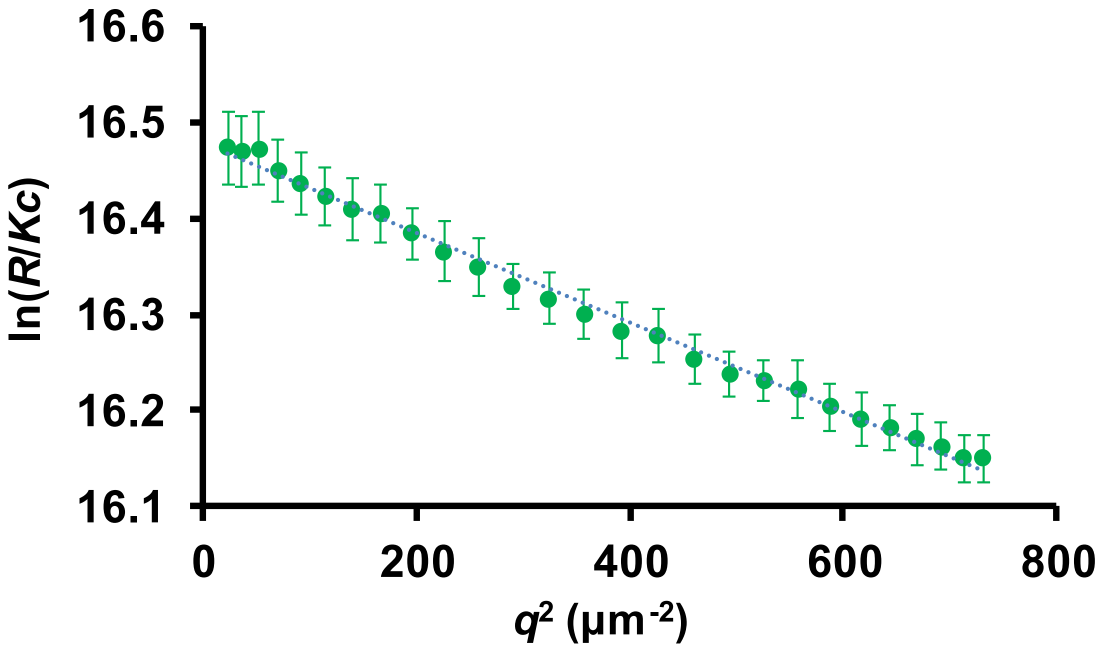

Supplementary Figure 15: Guinier plot, Ln(R/Kc) versus q^2, obtained by averaging the Guinier plots for five concentrations: 0.05, 0.18, 0.3, 0.6, and 1 mg mL^{-1}. The error bars represent the standard deviation of Ln(R/Kc) over the five concentrations. The equation of the linear trendline leads to a length of 115 nm, considering a radius cross-section, R_{SLS}, of 24 nm.

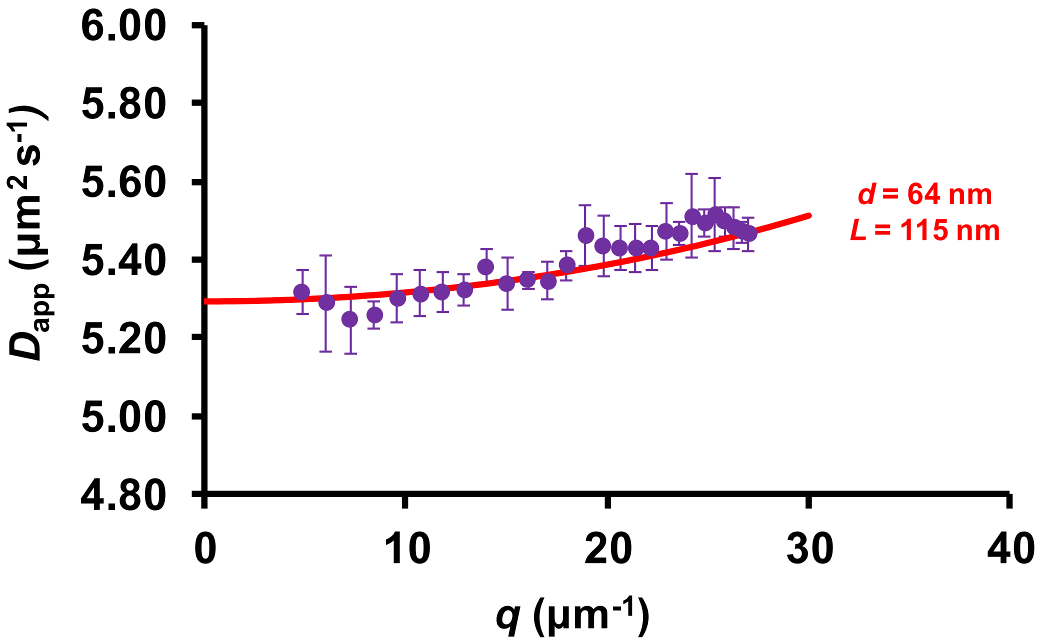

Supplementary Figure 16: Plot of D_{app} versus q, obtained by averaging D_{app} versus q calculated for five concentrations: 0.05, 0.18, 0.3, 0.6, and 1 mg mL^{-1}. The error bars represent the standard deviation of D_{app} over the five concentrations. To fit the data with Supplementary Equation 9, we fixed the value of L (115 nm from SLS) and used d as an adjustable parameter. The best fit was obtained for d = 64 nm

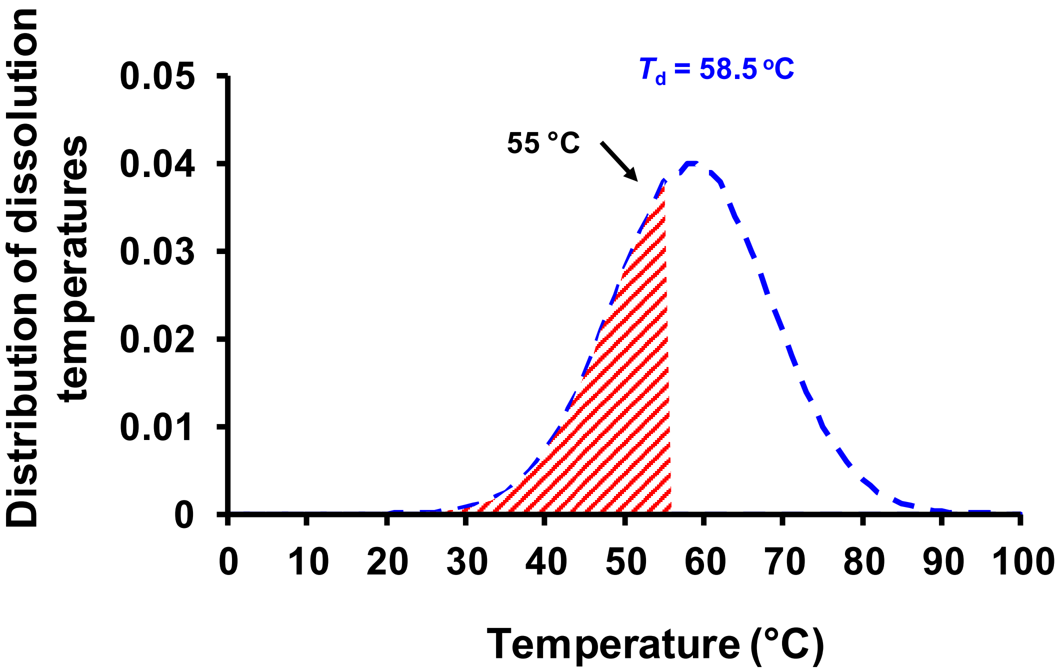

Supplementary Figure 17: Normal distribution of the dissolution temperatures of the PFS_{53}-*b*-PI_{637} seed crystallites. The average dissolution temperature is 58.5 °C. The fraction of seeds that dissolved at a given annealing temperature (for example 55 °C) is given by the area under the curve shown in red.

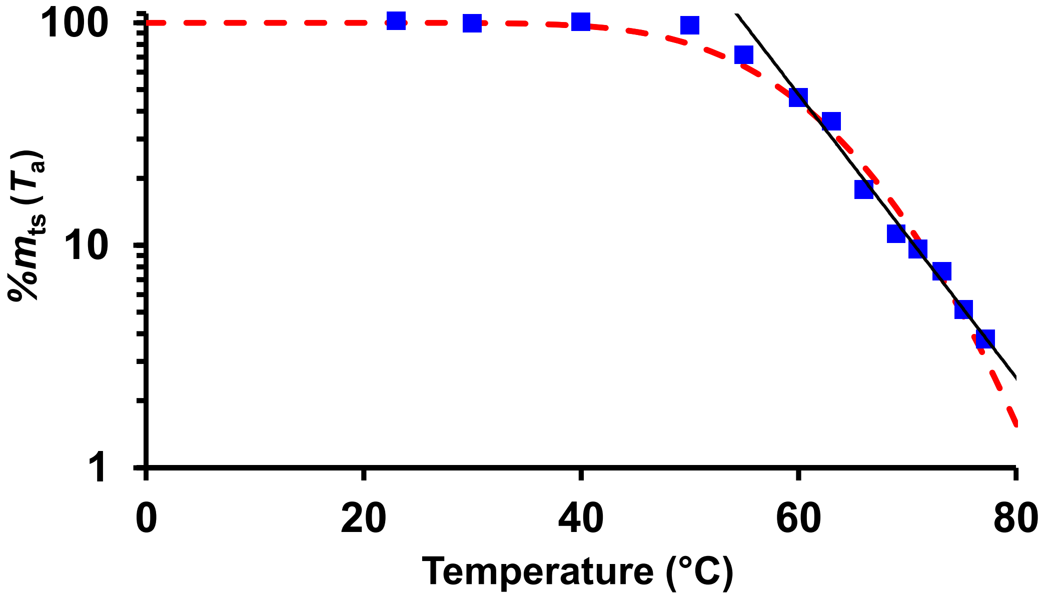

Supplementary Figure 18: Semilogarithmic plot of the mass percentage of surviving seeds in solutions of PFS_{53}-*b*-PI_{637} micelle fragments in decane vs annealing temperature. The red dashed line corresponds to the best fit obtained using Supplementary Equation 26. The black line represents the linear best fit for the 8 points of highest temperatures.[5] This linear line, however, fails to fit the data points at lower temperatures.

SUPPLEMENTARY REFERENCES

[1] Wilcoxon, J. & Schurr, J. M. Dynamic light scattering from thin rigid rods: Anisotropy of translational diffusion of tobacco mosaic virus. *Biopolymers* **22**, 849–867 (1983).

[2] Aragon, S. R. & Flamik, D. High precision transport properties of cylinders by the boundary element method. *Macromolecules* **42**, 6290–6299 (2009).

[3] Guerin, G., Qi, F., Cambridge, G., Manners, I. & Winnik, M. A. Evaluation of the cross section of elongated micelles by static and dynamic light scattering. *J. Phys. Chem. B* **116**, 4328–4337 (2012).

[4] Qian, J. *et al.* Self-seeding in one dimension: A route to uniform fiber-like nanostructures from block copolymers with a crystallizable core-forming block. *ACS Nano* **7,** 3754–3766 (2013).

[5] Qian, J. et al. Self-seeding in one dimension: an approach to control the length of fiberlike polyisoprene–polyferrocenylsilane block copolymer micelles. *Angew. Chem. Int. Ed.* **50**, 1622–1625 (2011).
